# Mechanistic Insight Into the Application of Alumina-Supported Pd Catalysts for the Hydrogenation of Nitrobenzene to Aniline

**DOI:** 10.1021/acs.iecr.2c01134

**Published:** 2022-07-14

**Authors:** Clément G. A. Morisse, Annelouise M. McCullagh, James W. Campbell, Chris Mitchell, Robert H. Carr, David Lennon

**Affiliations:** † School of Chemistry, Joseph Black Building, University of Glasgow, University Avenue, Glasgow G12 8QQ, U.K..; ‡ The Wilton Centre, SABIC UK Petrochemicals Ltd., Redcar, Cleveland TS10 4RF, U.K..; § Huntsman Polyurethanes, Everslaan 45, 3078 Everberg, Belgium

## Abstract

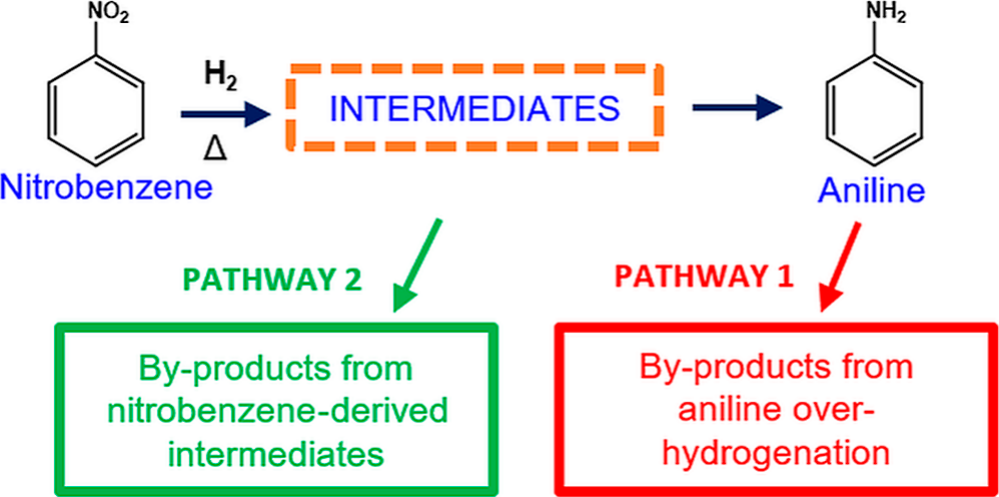

Two Pd/γ-Al_2_O_3_ catalysts are examined for the vapor phase hydrogenation of nitrobenzene over the temperature range of 60–200 °C. A 1 wt % catalyst is selected as a reference material that is diluted with γ-alumina to produce a 0.3 wt % sample, which is representative of a metal loading linked to a candidate industrial specification aniline synthesis catalyst. Cyclohexanone oxime is identified as a by-product that is associated with reagent transformation. Temperature-programed infrared spectroscopy and temperature-programed desorption measurements of chemisorbed CO provide information on the morphology of the crystallites of the higher Pd loading catalyst. The lower Pd loading sample exhibits a higher aniline selectivity by virtue of minimization of product overhydrogenation. Reaction testing measurements that were undertaken employing elevated hydrogen flow rates lead to the proposition of separate reagent and product-derived by-product formation pathways, each of which occurs in a consecutive manner. A global reaction scheme is proposed that defines the by-product distribution accessible by the grades of catalyst examined. This information is helpful in defining product purification procedures that would be required in certain heat recovery scenarios connected with large-scale aniline production.

## 1 Introduction

**Scheme 1:**
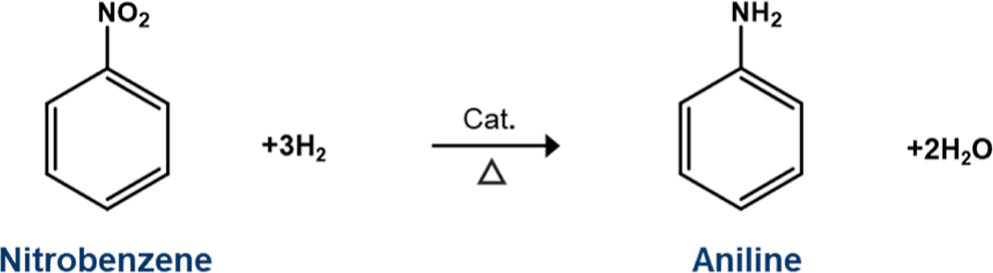
Hydrogenation of NB to Produce ANL

Aniline (ANL) is an important reagent within the chemical manufacturing sector. In 2008 the global ANL production was estimated to be 3.8 Mt, with ANL production capacity increasing beyond that time.^1^ A significant part of this production is used in the synthesis of isocyanates, mainly methylene diphenyl diisocyanate, which is used in the production of polyurethanes.^2^ A principal route for the large-scale production of ANL is via the hydrogenation of nitrobenzene (NB) over heterogeneous catalysts, Scheme 1, where liquid and vapor phase variants are employed.^1^ The reaction is highly exothermic, exhibiting standard reaction enthalpies of −554.1 and −468.2 kJ mol^–1^ for the liquid and vapor phases, respectively.^3^ Given that ANL synthesis unit operations are typically linked to isocyanate manufacturing facilities that constitute integrated chemical complexes,^4^ the NB hydrogenation reaction possesses excellent credentials for the application of heat recovery strategies to produce superheated steam for use throughout a chemical complex.^5^ Indeed, Päβler and Freund have described a model-based design of energy-efficient reactors for ANL synthesis that is intended to exploit the heat recovery possibilities this reaction affords.^6^

An issue in any intended heat recovery operation is that in order to produce superheated steam, the reaction needs to be operated at elevated temperatures (≥100 °C). This scenario can lead to complications in the catalytic conversion process, with elevated temperatures potentially compromising product selectivity via inadvertently providing access to chemical pathways that lead to by-product formation. As part of an initiative to improve the operational efficiency of certain ANL synthesis facilities associated with large-scale isocyanate production, the authors have recently considered the suitability of alumina-supported Pd catalysts for ANL synthesis operation at elevated temperatures.^5^ The matter of associating by-product formation with a specified catalyst formulation is a critical factor when considering the selection of a suitable postreaction purification stage (i.e., distillation unit) for any intended plant revisions at the industrial complex.

NB hydrogenation is comprehensively reported in the literature, with a wide number of catalysts examined.^7−11^ In 1898, Haber presented the first mechanism for ANL synthesis from NB hydrogenation using a Pt cathode for an electrochemical reduction reaction and proposed a three-step process to produce ANL: NB → nitrosobenzene (NSOB) → phenylhydroxylamine (PHA) → ANL.^7^ A series of azo compounds: azoxybenzene (AZOXY), azobenzene (AZO), and hydrazobenzene (HYDRAZO) were also included in the mechanism, emerging from the coupling of intermediates NSOB and PHA, with the decoupling of HYDRAZO reported to be a condensation route to ANL. This mechanism has wide acceptance in the realm of catalytic NB hydrogenation. In 2005, Gelder et al. supplemented this perspective by employing a series of hydrogenation/deuteration experiments over a Pd/C catalyst that excluded NSOB as an intermediate; leading them to propose a role for a Pd-hydroxyamino intermediate and the step-wise addition of hydrogen.^8^ Subsequent DFT investigations by Zhang et al. examining NB hydrogenation over a Pd_3_/Pt(111) bimetallic surface endorse a step-wise hydrogenation process via an adsorbed hydroxyamino intermediate.^12^

Both mechanisms hold great merit for understanding intermediates associated with NB hydrogenation to ANL; however, crucially important for commercial-scale ANL production, by-product formation arising from the in situ hydrogenation of ANL and various intermediate species [so-called “overhydrogenation” of NB], is also frequently reported.^13−19^ For example, with reference to the patent literature, Nagata et al. examined promoted Pd/C and Pd–Pt/C catalysts and reported the presence of cyclohexylamine (CHA) during the reaction, rationalizing its production via 2-steps: hydrogenation from ANL to an imine intermediate and then hydrogenation to CHA.^18^ Additionally, this imine intermediate is proposed to result in further by-products: cyclohexanone (CHO) through a hydration reaction and *N*-[1-(amino)cyclohexyl]-*N*-phenylamine (NPHA) via combination with ANL. In turn, both compounds eventually transform to produce *N*-cyclohexylaniline (CHAN) via different pathways. Moreover, CHO was reported to undergo coupling with ANL to give *N*-cyclohexylidenaniline (ANIL), which subsequently hydrogenates to give CHAN. NPHA can lead to CHAN directly via hydrogenation.^18^

Examining a series of alumina-supported Pd/Al_2_O_3_ catalysts, Couto et al. have developed these concepts further, additionally considering roles for benzene (BZ), cyclohexanol (CHOL), and dicyclohexylamine (DICHA) with the BZ originating via deamination of ANL.^16^ Couto et al. propose an amine intermediate to be active, not the imine reported by Nagata et al.,^18^ that leads to the formation of CHA, CHO, and NPHA. However, the mechanism by Couto et al. agrees with that of Nagata et al. concerning the coupling of CHO with ANL to give ANIL, Couto et al. present the production of CHOL via hydrogenation of CHO^16^ whilst Nagata et al. present CHAN as the final hydrogenation product.^18^ In contrast, Couto et al. depict DICHA to be the terminal molecule in the overhydrogenation pathway.

DICHA production is reported as a by-product throughout the literature; however, its origin is debated. There is some evidence that suggests that DICHA may be formed by self-coupling of CHA,^19^ but this instance was only observed for temperatures exceeding 160 °C. DICHA formation has also been proposed to occur via the coupling of CHA with an imine intermediate;^20^ Couto et al. suggest that DICHA formation is achieved simply by hydrogenation of CHAN.^16^

The documentary mentioned above reveals NB hydrogenation over supported metal catalysts to be intricate and to convey considerable complexity beyond that implied within Scheme 1. Indeed, investigations into the full nature of the formation of secondary by-products is an active area of research that has direct relevance to industrial operations. Following on from previous investigations from this group,^5^ this article is solely concerned with Pd/Al_2_O_3_ catalysts. Here, we identify another secondary by-product, cyclohexanone oxime (CHOX), which is associated with a chemical pathway involving NB-derived chemistry.

In addition to considering a mechanistic perspective for NB hydrogenation over Pd/Al_2_O_3_ catalysts at elevated temperatures, the article additionally seeks to link those deductions to a refined catalyst specification. Specifically, the morphology of the Pd crystallites, which can be evaluated by a combination of infrared (IR) spectroscopy and the chemisorption of CO as a probe molecule.^21^ Previous work from the authors examined two catalysts: a commercially available 5 wt % Pd/γ-Al_2_O_3_ catalyst (GU-1) and a 0.3 wt % Pd/γ-Al_2_O_3_ catalyst (GU-2). The work established that the low loading Pd/Al_2_O_3_ catalyst (0.3 wt % Pd) minimized the possibility of ANL overhydrogenation products—a favorable outcome for the industrial scenario, and one that guides specifications for a technical grade ANL synthesis catalyst.^5^ However, due to the low concentration of Pd surface atoms (∼4 μmol Pd_(s)_ g_(cat)_^–1^),^5^ these materials are difficult to analyze directly via the IR technique. To overcome this difficulty, a proof-of-principle experiment is outlined here in which the crystallite morphology of a low metal loading (0.3 wt % Pd) catalyst may be inferred from that of a higher metal loading sample. For this investigation, and to bridge the Pd concentration gap between 5.0 and 0.3 wt %, a commercially available 1 wt % Pd/γ-Al_2_O_3_ reference catalyst (GU-3) is utilized. Specifically, the 1 wt % Pd/γ-Al_2_O_3_ sample is diluted with γ-Al_2_O_3_ to produce a 0.3 wt % Pd/γ-Al_2_O_3_ catalyst (GU-4) that, thereby, possesses identical Pd crystallites to the reference “parent” catalyst, which is itself more amenable to investigation by IR spectroscopy. The low loading sample (GU-4) is then examined for NB hydrogenation so that outcomes observed can be correlated with Pd morphology, as determined for the 1 wt % Pd/γ-Al_2_O_3_ sample. These arrangements lead to the proposal for a global reaction scheme that applies to a catalyst specification suited to sustained ANL synthesis at temperatures that are compatible with possible reaction heat recovery operations at the industrial complex.

## 2 Experimental Section

A glossary of terms for all compounds given an abbreviation throughout the text is available in the Supporting Information section. Figure S1 presents a schematic representation of the apparatus used to perform reaction testing and infrared spectroscopic measurements of the catalysts under consideration.

### 2.1 Catalyst Preparation

Two catalysts are investigated in this study: a 1 wt % Pd/γ-Al_2_O_3_ catalyst supplied by Alfa Aesar (Ref: 11711) and a 0.3 wt % Pd/Al_2_O_3_ catalyst. The 1 wt % Pd/γ-Al_2_O_3_ powder catalyst was investigated as received. XRD analysis of the 1 wt % Pd/Al_2_O_3_ catalyst confirmed the support material to be γ-alumina^22^ (Figure S2a). Subsequently, the 0.3 wt % Pd/Al_2_O_3_ catalyst was prepared via mixing of 1 wt % Pd/γ-Al_2_O_3_ (9 mg) with γ-alumina (XRD: Figure S2b) (21 mg, Ineos, Ref: 25867/18A)^23^ to give the desired metal loading. No additional treatments were performed. The resulting 0.3 wt % catalyst contained the exact same Pd crystallites as the “parent” sample but at a lower concentration than the more IR amenable higher loading reference material. From here on in, the 1 wt % Pd/γ-Al_2_O_3_ and 0.3 wt % samples will be referred to as GU-3 and GU-4, respectively. Table S1 summarizes the nomenclature of terms used to explain the five distinct Pd/Al_2_O_3_ catalysts examined in this study and in two preceding articles.^5,21^

### 2.2 Characterization

#### 2.2.1 XRD, CO Chemisorption, and AAS

Powder X-ray diffraction (XRD) was performed with a Rigaku MiniFlex diffractometer (source accelerating voltage: 40 kV; source intensity: 15 mA) using Cu Kα (1.5406 Å) radiation (range: 5–80° θ). XRD patterns were monitored using a scan rate of 1° s^–1^.

CO adsorption isotherms obtained at 25 °C employed a pulse-flow method utilizing an in-line gas chromatograph (Thermo Finnigan Ultra GC, Trace GC, TCD detector) to determine the chemisorption capacity of both catalysts. Assuming a surface stoichiometry of CO/Pd = 1:2,^24^ these values were used further to estimate Pd dispersion and mean particle size.

Palladium loading was measured by atomic absorption spectroscopy (AAS) using a Perkin Elmer analyst 100 instrument (λ = 244.8 nm) that was calibrated from a 1 g L^–1^ Pd/HCl commercial stock solution (Sigma Aldrich). Samples were prepared for analysis by dissolving the catalyst sample (0.1 g) in aqua regia, boiling for 30 min allowing fumes to evaporate. After cooling, deionized water (5 mL) was added and the solution was filtered into a 25 mL volumetric flask.

#### 2.2.2 CO Temperature-Programed IR (1 wt % Pd/Al_2_O_3_, GU-3)

Diffuse reflectance infrared Fourier transform spectroscopy (DRIFTS) was performed in situ with a Nicolet Nexus FTIR spectrometer fitted with a SpectraTech Smart diffuse reflectance cell and an environmental chamber. The as-received GU-3 sample was reduced in the ceramic sample cup (*ca.* 30 mg) in a flow of He (BOC gases, 99.9%) and H_2_ (BOC gases, 99.8%), while heated to 110 °C, and held at this temperature for 30 min. The temperature was increased to 200 °C for 1 h, and the H_2_ flow was stopped after 30 min. The sample was permitted to return to ambient temperature in flowing He, and a background spectrum was recorded. The sample was exposed to CO (CK gases, 99.99%) and subsequently flushed with He to remove nonchemisorbed CO from the sample chamber. Spectra were recorded at 27 °C (520 scans at 4 cm^–1^ resolution). For desorption measurements, the catalyst was heated in situ under a He flow and maintained at the selected temperature for 10 min before cooling to 27 °C for spectral acquisition. This process was repeated at 50, 100, 150, 200, 250, 300, and 350 °C. Spectra are presented as difference spectra, where the spectrum of a clean, activated catalyst has been subtracted from that of a CO-dosed spectrum. No additional spectral manipulation was performed. The surface morphology of GU-4 can be inferred from CO TP-IR measurements performed on GU-3. Both samples contain identical Pd crystallites due to the sample dilution process, which provides the opportunity to evaluate the morphology of low-loading Pd/Al_2_O_3_ samples.^5^

#### 2.2.3 CO Temperature-Programed Desorption (1 wt % Pd/Al_2_O_3_, GU-3)

Activation of the catalyst (500 mg) within a stainless-steel reactor utilized a flow of He/H_2_ (35/15 mL min^–1^) and a temperature ramp (5 °C min^–1^) up to 200 °C. The temperature was held for 1 h with H_2_ flow stopped after 30 min. The sample was exposed to CO (CK gases, 99.99%) and, subsequently, flushed with He to remove nonchemisorbed CO. For desorption, a temperature ramp (15 °C min^–1^) to a final temperature of 600 °C was used under a flow of helium (35 mL min^–1^). Desorbed species were analyzed via mass spectrometry (MKS Microvision plus).

### 2.3 Reaction Testing

Two reaction testing regimes were explored. A baseline H_2_/C_6_H_5_NO_2_ molar flow ratio of 40:1 constituted the vast majority of experiments. However, in order to explore issues connected with surface hydrogen supply, a small number of measurements were performed under conditions of an excess hydrogen regime. Here an H_2_/C_6_H_5_NO_2_ molar flow ratio of 600:1 was adopted. Selectivity profiles for the excess hydrogen regime are presented in Sections 3.2.1 and 3.2.2. The following gas flows were utilized to achieve the desired H_2_/C_6_H_5_NO_2_ molar flow ratios: 40:1 = H_2_: 1.3 mL min^–1^; He: 33.7 mL min^–1^; 600:1 = H_2_: 12.5 mL min^–1^; He: 22 mL min^–1^.

With reference to Figure S1, reaction testing was carried out in the vapor phase via a 1/4 inch 500 mm continuous plug flow reactor (1/4” Swagelok, internal diameter: 0.18″) housed in a split tube furnace (LPC Elements, DMG control) with a *ca.* 30 mg catalyst charge. Activation of catalysts was performed in situ as described in Section 2.2.3. Hydrogen (BOC gases, 99.8%) and helium (BOC gases, 99.9%) were supplied by mass flow controllers (Brooks, 5850 TR). NB was supplied as a vapor using a heated bubbler set-up that delivered 1.08 μmol(NB) min^–1^ for H_2_/C6_5_H_5_NO_2_ = 40:1 and 1.00 μmol(NB) min^–1^ for H_2_/C_6_H_5_NO_2_ = 600:1, with a standard deviation-derived error of ±3.5%; weight hourly space velocity (WHSV) values equated to 0.27 and 0.20 h^–1^ for low and high hydrogen regimes, respectively. All gas lines leading to and exiting the reactor were kept at a fixed temperature (60 °C) using heating tape (Electrothermal, HT95508) controlled via a Eurotherm P818 Process Controller to ensure that compounds were retained in the vapor phase. Analysis was carried out using gas–liquid chromatography via an Agilent 6850 series II instrument fitted with a Durabond DB-17 capillary column (30 m, 0.250 mm, 0.5 μm) and an FID detector. GLC samples were taken using a 250 μL gas-sampling valve. A 40 h catalyst conditioning phase was utilized, in which NB hydrogenation was run at 60 °C to allow the reaction to stabilize prior to data collection. Replicate data points were collected under steady-state conditions for the following temperatures: 60, 100, 140, 180, and 200 °C, with data presented as an average value.

NB conversion was calculated according to eq 1(25)
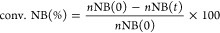
1where *n*NB(0) represents the initial number of moles of NB, and *n*NB(*t*) represents the number of moles of NB at time *t*. Product selectivity values were calculated according to eq 2(25)
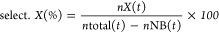
2where *nX*(*t*) represents the number of moles of compound *X* at time *t*, and *n*total(*t*) represents the total number of moles of all observed compounds at time *t*. WHSV values, defined as the mass of NB normalized to catalyst mass per unit time,^26−28^ were calculated according to eq 3
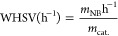
3Where *m*_NB_ h^–1^ represents the mass of the NB per hour, and *m*_cat._ represents catalyst mass.

### 2.4 Identification of CHOX as a By-product

Previously, the hydrogenation of NB to ANL using an industrial-grade 0.3 wt % Pd/Al_2_O_3_ catalyst signified as GU-2^5^ exclusively permitted observation of an unknown, low quantity by-product that exhibited a retention time in GC chromatograms between those of ANL and NB—an indication that the by-product possessed a molecular weight/boiling point intermediary between that of NB (mol. wt.: 123.1 g mol^–1^, B.Pt. = 210 °C) and ANL (mol. wt.: 93.1 g mol^–1^, B.Pt. = 184 °C). This unknown by-product is identified herewith via the experimental protocol outlined below.

The low quantity of the unknown by-product from reaction over GU-2 hampered its identification. However, a different 0.3 wt % Pd/Al_2_O_3_ technical grade egg-shell catalyst supplied by Huntsman Polyurethanes (Ref: ASC-2) happened to produce the unknown material in significantly greater quantities than that observed with GU-2. This catalyst is designated as GU-5 (Table S1) and was applied in this investigation as a means for unknown identification only. Characterization details for GU-5 are presented in the Supporting Information section (Table S2: AAS, CO adsorption isotherm, BET surface area). We have previously fractionated the by-product distribution for NB hydrogenation over Pd/Al_2_O_3_ as comprising two principal pathways: pathway-1 (major) corresponds to overhydrogenation of ANL, and pathway-2 (minor) relates to by-products formed from the transformation of NB; we now associate the unknown compound with pathway-2.^5^Figure S3 presents the selectivity of NB hydrogenation by-products as a function of increasing temperature on reaction over GU-5 after a 16 h conditioning phase at 60 °C. At a reaction temperature of 60 °C, the unknown is the only by-product detected. On increasing the temperature to 100 °C, the unknown by-product is no longer observable and, instead, DICHA is uniquely observed. The concentration of this entity increases as a function of temperature. At 140 °C a small contribution of CHAN is seen, which also increases as a function of temperature. Thus, GU-5, a 16-h catalyst conditioning period, and a reaction temperature of 60 °C were selected to facilitate NB hydrogenation that enabled adequate quantities of the unknown by-product to be produced and subsequently analyzed.

A cryogenically cooled trap was inserted in the reactor exit stream and reaction testing at 60 °C was performed similarly to that described in Section 2.3 for 48 h. The resulting condensate was isolated and analyzed by GC–MS using a Shimadzu GC-MS-QP2010S model coupled to a Shimadzu GC-2010 equipped with a ZB-5MS capillary column (30 m × 0.25 mm × 0.25 μm). The mass spectrum of the eluent corresponding to the retention time of the unknown by-product is presented in Figure S4; Table S3 presents the assignments for the observed fragmentation pattern. With a parent *m*/*z* value of 113 amu, and with reference to the mass spectrometer database,^29^ the molecule is assigned to CHOX (C_6_H_10_NOH, B.Pt. = 205 °C). To the best knowledge of the authors, this is the first time that this molecular entity has been identified as a by-product in NB hydrogenation over Pd/Al_2_O_3_ catalysts without the addition of any hydroxylamine species to the feed. The mechanistic relevance of CHOX to pathways accessible in NB hydrogenation will be considered in the Discussion section.

## 3 Results

Henceforth, results refer to GU-3 and GU-4 exclusively.

### 3.1 Catalyst Characterization

#### 3.1.1 CO Chemisorption and AAS

Table 1 summarizes catalyst characterization measurements. AAS measurements provided Pd loadings for GU-3 and GU-4 of 0.92 wt % and 0.32 wt %, respectively, confirming that the desired metal loading of 0.3 wt % Pd was achieved for GU-4 via dilution of GU-3 with γ-alumina. CO chemisorption results revealed the metal dispersion and estimated mean particle size for both catalysts to be the same (41 vs 38% and 2.53 vs 2.95 nm ± 10%, respectively). Note that the variances in estimated mean particle size between GU-3 and GU-4 represent the error associated with repeated quantification of saturation points as determined by CO adsorption isotherms. Therefore, as intended, GU-4 is comprised of Pd crystallites of comparable dimensions to GU-3 but at a lower density of metal, which is more representative of industrial catalyst loading specifications. As anticipated, the concentration of surface Pd atoms, [Pd_(s)_], does vary between the catalysts, GU-3/GU-4 = 3.4:1.0, and is attributed to the differences in metal loading. Thus, this methodology now enables comparisons on the effect of metal concentration on catalytic activity to be assessed for a fixed Pd crystallite morphology, where the morphology of the lower Pd loading catalyst (GU-4) can be inferred with due reference to the higher Pd loading sample (GU-3).

**Table 1:** Metal Weighting (AAS), Dispersion, Particle Size, and Surface Pd Concentration of GU-3 and GU-4^a^

sample	nominal loading (%)	weighting (%)	dispersion (%)	particle size (nm)	Pd surface (μmol g^–1^)
GU-3	1	0.92 ± 0.045	41	2.53 ± 0.253	38.6
GU-4	0.3	0.32 ± 0.018	38	2.95 ± 0.295	10.7

a The latter three parameters are determined from CO adsorption isotherm measurements

#### 3.1.2 CO TP-IR (GU-3)

**Figure 1:**
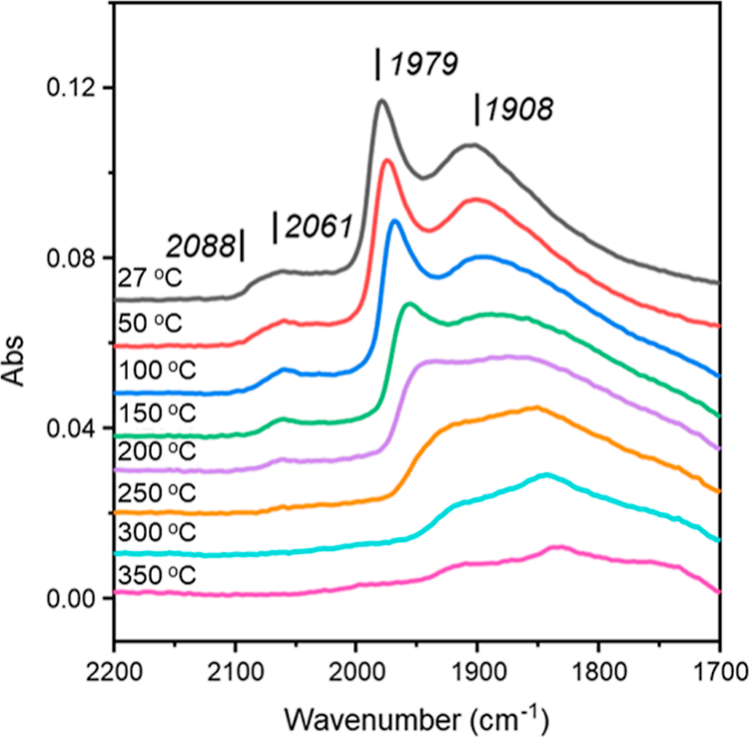
CO TP-IR DRIFTS spectra of GU-3. Spectra have been offset by 0.01 a.u. to facilitate viewing.

DRIFTS of chemisorbed CO has been utilized previously to investigate a 0.3 wt % Pd/Al_2_O_3_ catalyst (GU-2). However, analysis of desorption temperatures exceeding 200 °C resulted in inferior signal-to-noise ratios.^5^ Characterization of the surface morphology of the higher loading GU-3, with the same Pd nanoparticle structure as GU-4, will, in turn, permit an understanding of the surface morphology of the low weighting catalyst and is intended to circumnavigate this issue. Figure 1 gives the CO TP-IR DRIFTS spectra collected using the higher metal loading catalyst, GU-3. The spectrum collected at ambient temperature exhibits 4 spectral features, the most intense of which are two broad peaks centered at 1979 and 1908 cm^–1^ corresponding to adsorption of μ_2_ bridge-bonded CO on Pd(100) planes and μ_3_ bridge-bonded CO on Pd(111) planes, respectively.^21,30^ Additionally, a lower intensity broad peak at 2061 cm^–1^ is assigned to linear CO adsorption to edge sites, whilst a small shoulder at 2088 cm^–1^ is assigned to linear CO adsorption on corner sites.^30^ The 27 °C spectrum of CO adsorbed over GU-3, and derivation of 2 distinct linear CO adsorption sites, closely matches that of previous CO TP-IR measurements over alumina-supported palladium catalysts^5,21,30^ and is indicative of the Pd crystallites adopting a truncated cubo-octahedral structure.^30^

The peak intensity of all features reduced as temperatures were incrementally elevated. The feature assigned to linear CO adsorption on Pd corner sites at 2088 cm^–1^ was observable up to 100 °C, whereas linear CO_(ad)_ on edge sites was retained on the catalyst surface up to 250 °C. Both μ_2_ and μ_3_ bridge-bonded CO remained present on GU-3 for the maximum temperature investigated (350 °C). Therefore, CO_(ad)_ binding strength to GU-3 exhibited the following trend: bridge-bonded CO (μ_2_ and μ_3_) > linear CO (edge) > linear CO (corner). These trends agree with those reported previously for CO_(ad)_ over 5 wt % Pd/γ-Al_2_O_3_ (GU-1)^5,21^ and 0.3 wt % Pd/Al_2_O_3_ (GU-2).^5^

Some peak shifting was observed as a function of increasing temperature for peaks assigned to bridge-bonded CO_(ad)_. Significant shifts from 1979 to 1910 and 1908 to 1833 cm^–1^ were observed for μ_2_ and μ_3_ bridge-bonded CO, respectively. These shifts result from lateral interactions between neighboring molecules due to dipole coupling effects, which reduce with decreasing surface coverage.^31^

As described in Section 2.2.2, CO TP-IR-derived insights into GU-3 Pd crystallites are extendable to GU-4, the lower loading sample. Thus, it is deduced that GU-4 may also be described as possessing Pd crystallites that exhibit a truncated cubo-octahedral structure.

#### 3.1.3 CO TPD (GU-3)

**Figure 2:**
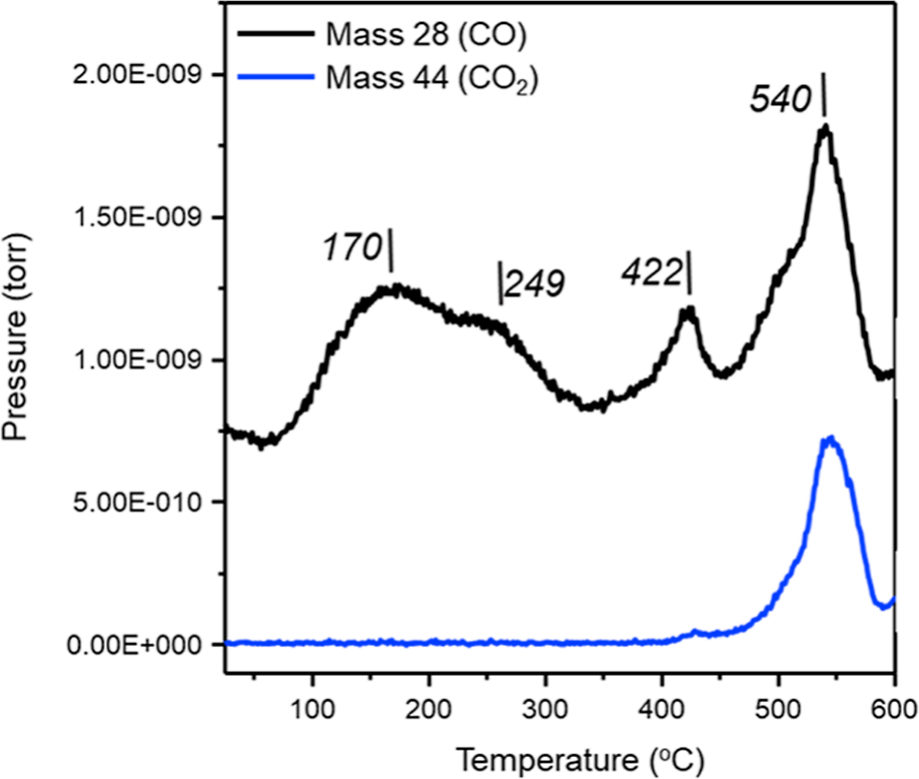
CO TPD of GU-3 showing the evolution of CO (mass 28, black) and CO_2_ (mass 44, blue) as a function of increasing temperature up to 600 °C.

Linking to the abovementioned discussion of CO TP-IR spectra, CO temperature-programed desorption (TPD) results for GU-3 (Figure 2) reveal a range of CO adsorption sites on Pd; peak maxima are observed at 170, 249, 422, and 540 °C. The 170 °C peak is thought to have contributions from linear CO (corner and edge), the 249 °C peak mainly indicates desorption from linear CO (edge) and μ_2_ CO, whilst the 422 °C peak is attributed to desorption from bridge-bonded CO (μ_2_ and μ_3_). CO desorption peaks for temperatures <300 °C are generally accepted as arising from linearly bound CO, and peaks for temperatures >300 °C, from bridging CO.^32−35^ In general, CO desorption from GU-3 followed this trend.

The CO peak at 540 °C in Figure 2 is not assigned to CO desorption; rather, it is associated with CO_2_ desorption, with the CO contribution representing a fragment of the CO_2_ mass spectrum. Isotopic substitution experiments for CO adsorption on Pd/Al_2_O_3_ catalysts performed by Lear et al. similarly exhibit a high-temperature CO_2_ desorption feature, which is attributed to the decomposition of alumina carbonate groups present in the metal/support interface;^36^ this is thought to be the origin of the intense CO_2_/CO feature in Figure 2.

### 3.2 Reaction Testing

**Figure 3:**
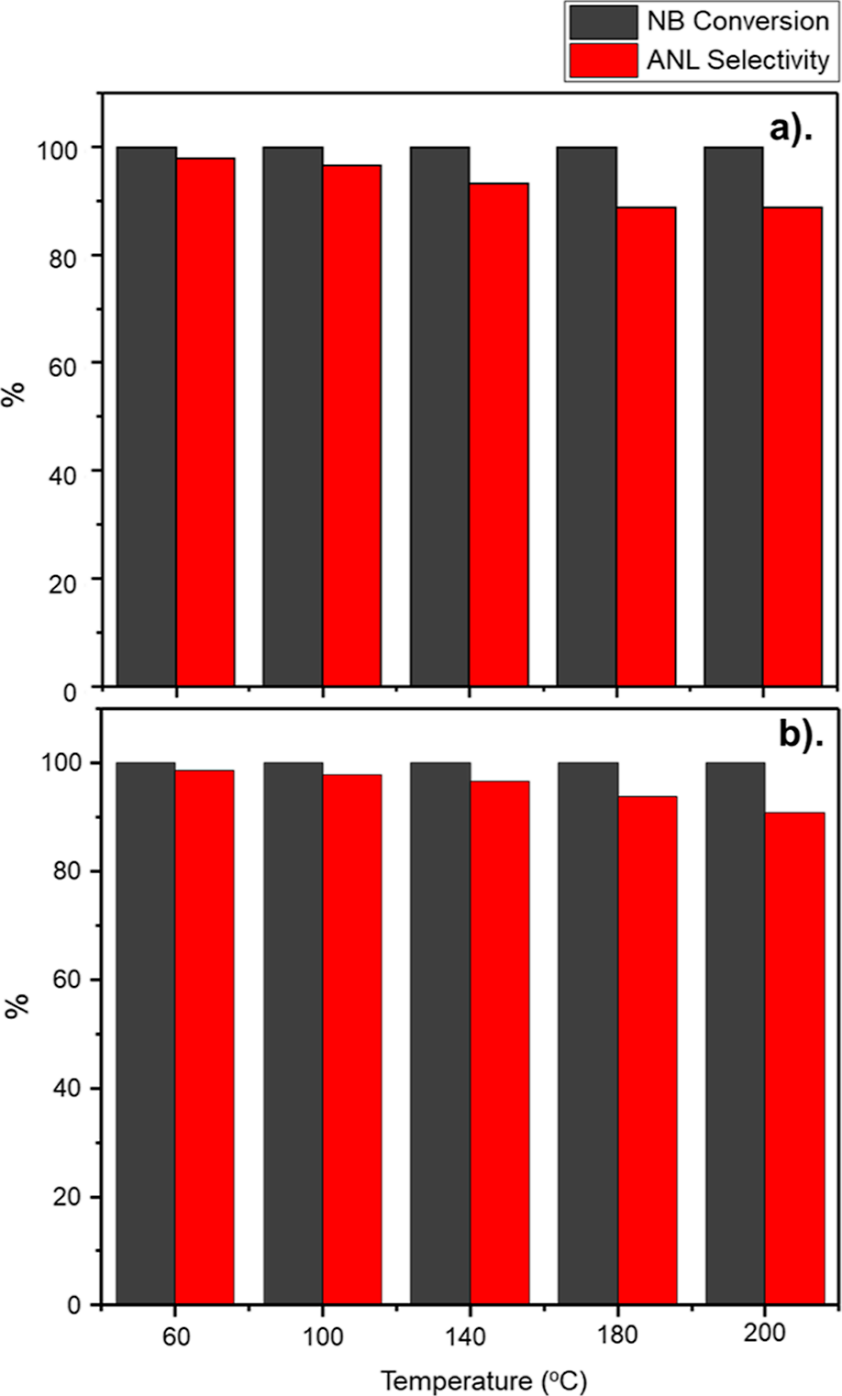
NB conversion (grey) and ANL selectivity (red) as a function of reaction temperature (standard hydrogenation conditions: H_2_/C_6_H_5_NO_2_ molar flow ratio = 40:1 and WHSV = 0.27 h^–1^): (a) GU-3 and (b) GU-4.

Figure 3 presents the NB conversion (grey) and ANL selectivity (red) values for GU-3 (a) and GU-4 (b) as a function of temperature for the baseline hydrogen regime (H_2_/C_6_H_5_NO_2_ molar flow ratio = 40:1) and portrays nearly complete NB conversion (GU-3 ≥ 99.86%; GU-4 ≥ 99.99%) irrespective of catalyst choice. Operation at full NB conversion is representative of operations in the industrial scenario.^1^ As illustrated in Figure 3, in both cases, the organic flux is not overloading the number of active sites presented by the catalysts. Blank reaction testing on a reference γ-alumina sample revealed minimal NB conversion; therefore, it is deduced that all the hydrogenation activity is attributed to the presence of the Pd nanoparticles for GU-3 and GU-4.

Figure 3 shows ANL selectivity to exhibit the same general trend for both GU-3 and GU-4—a decrease in ANL selectivity with increasing temperature, but exact ANL selectivity values differed. As anticipated, ANL selectivity was optimized for both catalysts at 60 °C, yielding selectivity values of 97.7 and 98.7% for GU-3 and GU-4, respectively. On increasing reaction temperature to 200 °C ANL selectivity decreased to 88.6% for GU-3 and 91.0% for GU-4; confirming the trend that alongside a relatively low concentration of hydrogen (H_2_/NB = *ca.* 40:1), low Pd loadings facilitate favorable ANL yields at the elevated temperatures associated with possible plant heat recovery strategies.^5^

#### 3.2.1 ANL-Derived By-products (Pathway-1)

**Figure 4:**
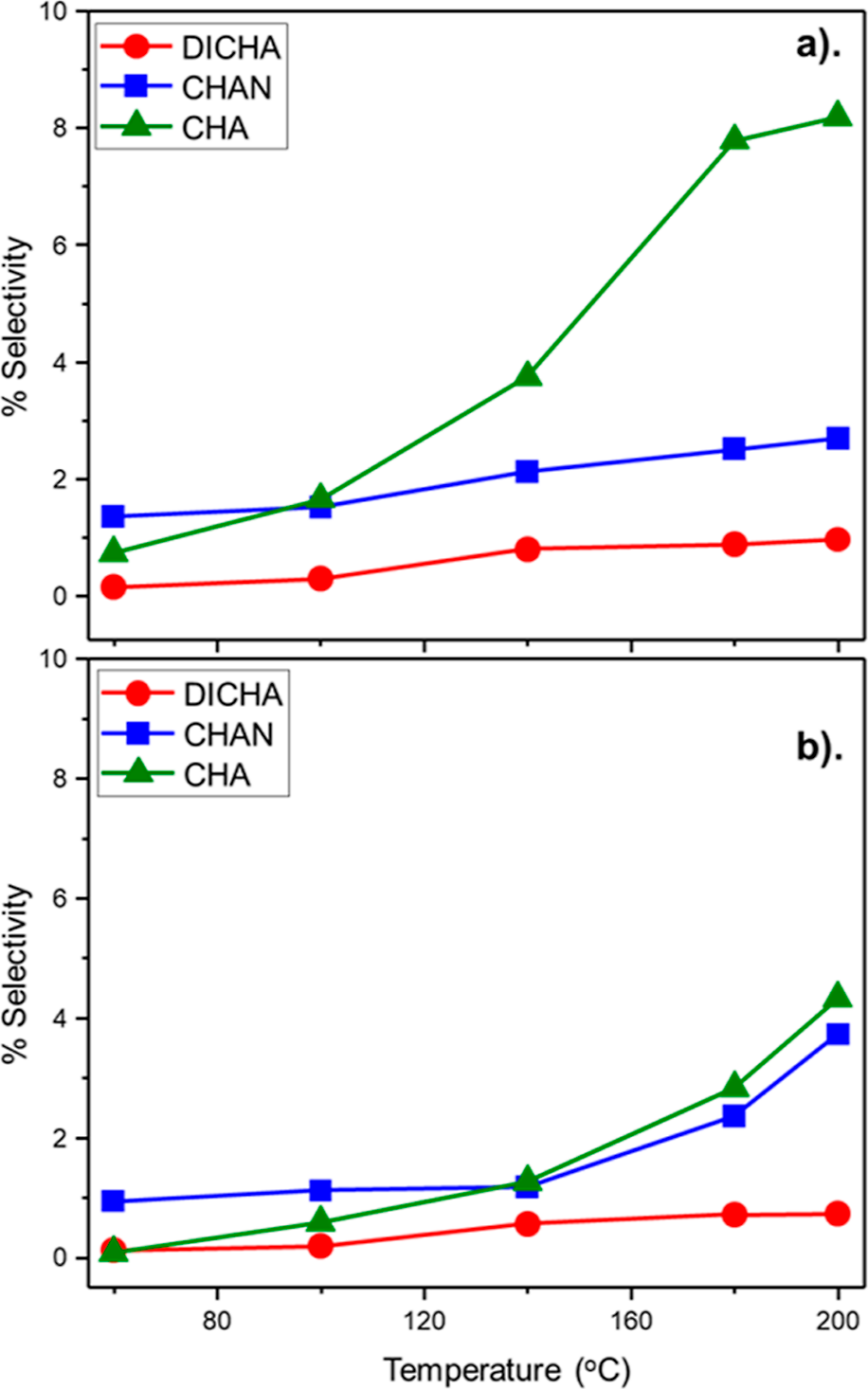
Selectivity of ANL overhydrogenation by-products [DICHA, CHAN, and CHA] as a function of temperature: (standard hydrogenation conditions: H_2_/C_6_H_5_NO_2_ molar flow ratio = 40:1 and WHSV = 0.27 h^–1^): (a) GU-3 and (b) GU-4.

Figure 4 presents by-products arising from the overhydrogenation of ANL (CHA, CHAN, and DICHA) as a function of temperature for GU-3 (a) and GU-4 (b) operating within the baseline hydrogen regime (H_2_/C_6_H_5_NO_2_ molar flow ratio = 40:1). Selectivity for DICHA remained relatively low throughout the testing of both catalysts, reaching a maximum of 1.0 and 0.7% for GU-3 and GU-4, respectively. This outcome is thought to reflect hydrogen constraints within the reaction system that limits the hydrogenation of CHAN to DICHA. At elevated temperatures, CHA is the dominant by-product for both catalysts, although the value for GU-3 (*S*_CHA_ = 8.1%) is approximately double that seen for GU-4 (*S*_CHA_ = 4.1%). Given that the incident hydrogen flow and the Pd crystallite morphology are identical, we propose that GU-3’s greater concentration of surface sites (38.6 μmol Pd_(s)_ g_(cat)_^–1^, Section 3.1.1) leads to an enhanced supply of chemisorbed hydrogen atoms that support ring hydrogenation of ANL to form CHA. However, at a H_2_/NB molar flow ratio of 40:1, there is seemingly insufficient surface hydrogen for significant further hydrogenation. Conversely, the lower concentration of Pd surface sites on GU-4 (10.7 μmol Pd_(s)_ g_(cat)_^–1^) corresponds to a more constrained supply of surface hydrogen, and hence, lower quantities of CHA are observed.

**Figure 5:**
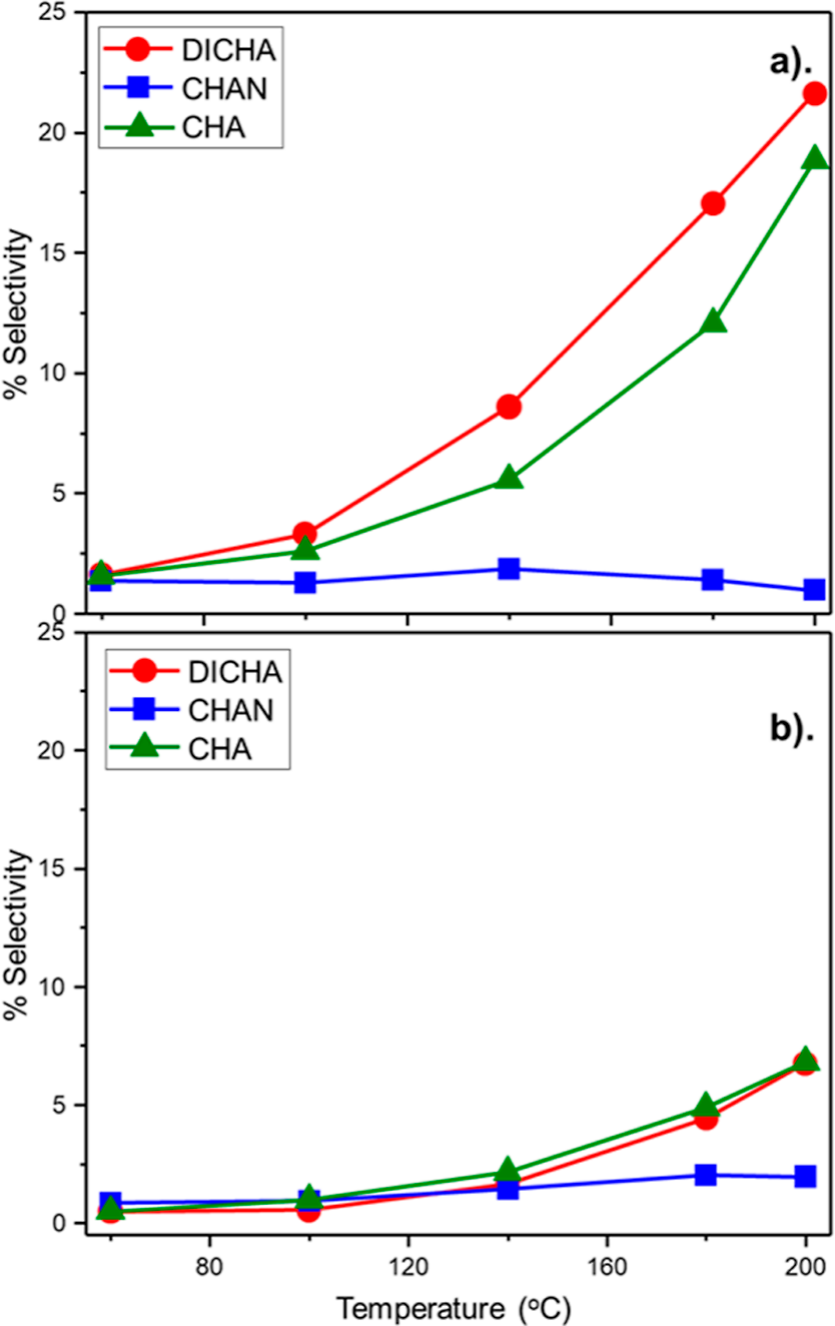
ANL overhydrogenation by-products DICHA, CHAN, and CHA as a function of temperature in the presence of an enhanced flow of dihydrogen: (H_2_/C_6_H_5_NO_2_ molar flow ratio = 600:1 and WHSV = 0.20 h^–1^): (a) GU-3 and (b) GU-4.

To explore further the concept of hydrogen supply, additional reaction testing was undertaken utilizing an elevated hydrogen regime (H_2_/C_6_H_5_NO_2_ molar flow ratio = 600:1; Section 2.3). Under this regime NB conversion remained near completion; however, ANL selectivity significantly decreased as a function of both metal loading and temperature (Figure S5). Figure 5 presents the ANL-derived by-product selectivity values and reveals increased yields of CHA and DICHA, yet the levels of CHAN are comparable to that observed in the lower hydrogen regime (H_2_/C_6_H_5_NO_2_ molar flow ratio = 40:1, Figure 4).

**Scheme 2:**
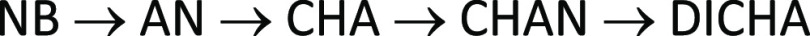
Proposed Stepwise Hydrogenation Pathway for ANL-Derived By-products^5^

This outcome signifies rapid hydrogenation of CHAN to DICHA in the excessive hydrogen regime, such that levels of detectable CHAN were constrained. Consideration of data sets for GU-3 and GU-4 acquired with low (Figure 4) and excess (Figure 5) hydrogen regimes are consistent with a stepwise hydrogenation process, as illustrated in Scheme 2. This agrees with the stepwise process reported for ANL-derived by-products in an investigation by Corma et al., who investigated the liquid phase hydrogenation of CHA over Pd/C and yielded DICHA;^37^ confirming the production of DICHA from a consecutive process originating from CHA and not from an imine^18^ or amine^16^ intermediate as reported elsewhere.

#### 3.2.2 NB-derived By-products (Pathway-2)

**Figure 6:**
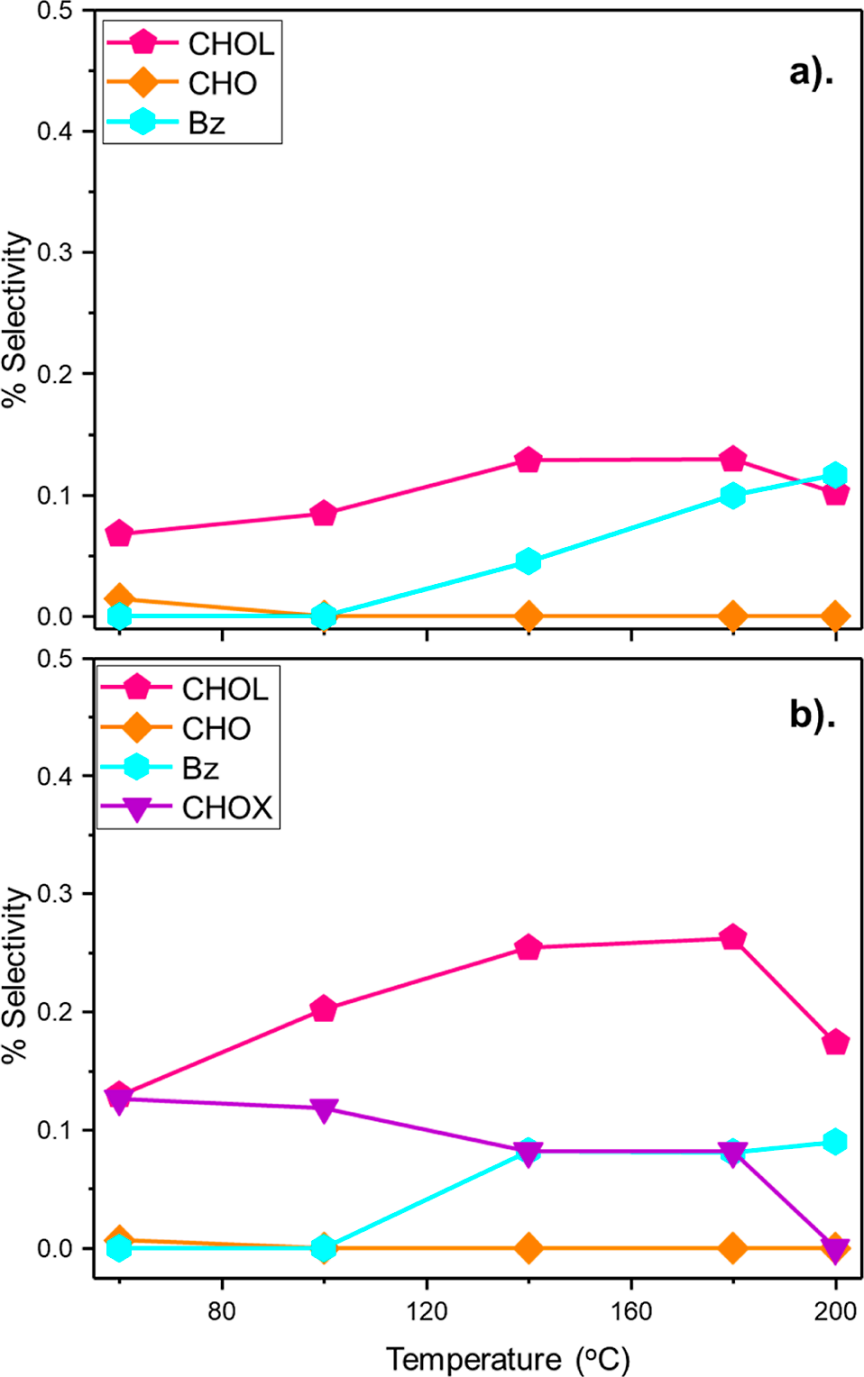
Selectivity of NB-derived by-products [CHOL, CHO, CHOX] and BZ as a function of temperature: (standard hydrogenation conditions: H_2_/C_6_H_5_NO_2_ molar flow ratio = 40:1 and WHSV = 0.27 h^–1^): (a) GU-3 and (b) GU-4.

Reverting to the baseline testing regime of a H_2_/C_6_H_5_NO_2_ molar flow ratio = 40:1, low (selectivity {S} < 0.5%) quantities of Pathway-2 by-products [CHO, CHOL] and BZ were identified for GU-3 (Figure 6a) and GU-4 (Figure 6b). Trends concerning these by-products are comparable for both catalysts. CHO was observed at < 0.02% at 60 °C for both GU-3 and GU-4 and was not observable for reaction temperatures exceeding this value—an indication of the increased hydrogenation ability of the catalysts to transform CHO to CHOL with increasing temperature. Consequently, *S*_CHOL_ increased with increasing temperature up to a maximum of 0.12% for GU-3 and 0.26% for GU-4 at 180 °C, after which quantities of CHOL decreased. BZ was detected for temperatures ≥140 °C; its presence is thought to be indicative of activated deamination of ANL.^16^

NB hydrogenation with GU-4 also yielded the previously unknown by-product^5^ CHOX for temperatures up to 180 °C. CHOX demonstrated a maximum selectivity value of 0.12% at 60 °C that progressively decreased at elevated temperatures. No CHOX was detected for GU-3. This outcome is unusual, and while an exact argument for CHOX formation cannot be presented here, the observation of CHOX with GU-4 and not GU-3 may be rationalized with respect to a surface-hydrogen-supply issue that plays a critical role in modifying product distributions within the reaction system. As abovementioned, given that the incident hydrogen flow and the Pd crystallite morphology are identical, we propose that GU-3’s greater concentration of surface sites leads to an enhanced supply of chemisorbed hydrogen atoms that favors hydrogenation of CHOX → CHO. Conversely, the lower concentration of Pd surface sites on GU-4 corresponds to a more constrained supply of surface hydrogen and, hence, lower CHOX hydrogenation rates. Thus, CHOX is only observable with GU-4, as is the case experimentally (Figure 6b).

**Figure 7:**
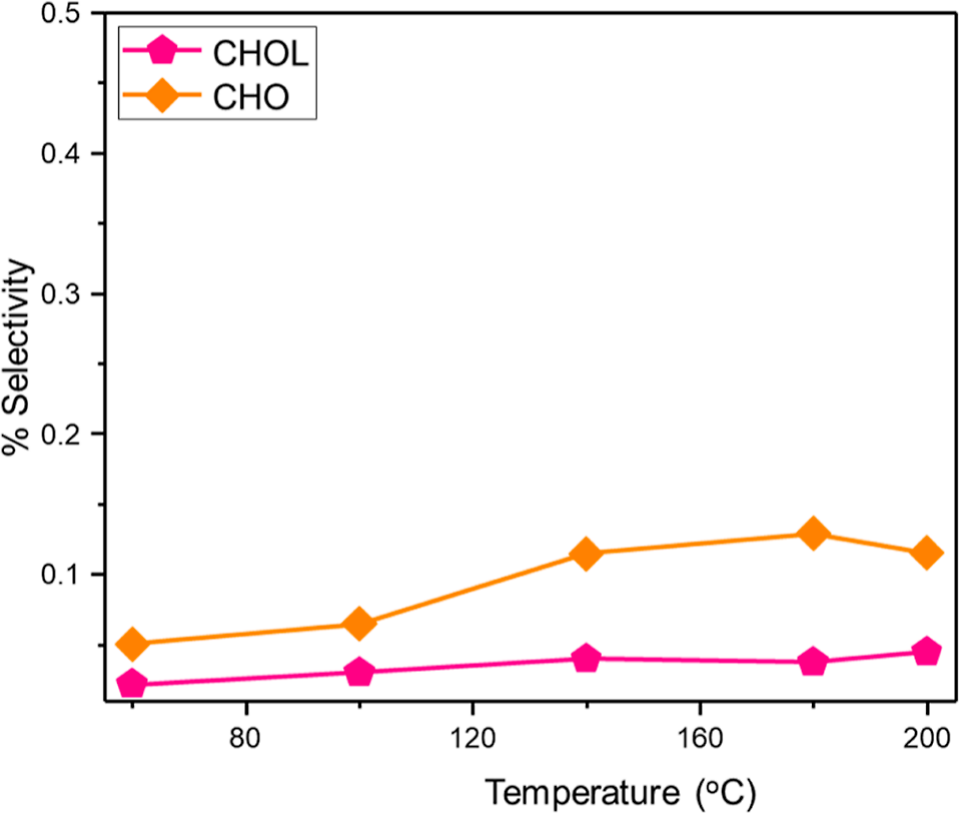
NB-derived by-products CHOL and CHO as a function of temperature for GU-4 in the presence of an enhanced flow of dihydrogen: (H_2_/C_6_H_5_NO_2_ molar flow ratio = 600:1 and WHSV = 0.20 h^–1^).

Figure 7 considers the case for NB-derived by-products for the excess hydrogen regime (H_2_/C_6_H_5_NO_2_ molar flow ratio = 600:1 and WHSV = 0.20 h^–1^) for GU-4. Now CHOX is not detected and a maximum CHOL selectivity of 0.13% is observed, which contrasts with the low hydrogen regime measurements (*S*_CHOL_ = 0.26%, Figure 6b). This outcome is suggestive that the formation of NB-derived by-products is more favored under conditions of constrained hydrogen supply and that this pathway is in a competitive regime with the primary reaction, that is, NB → ANL. This nonobservation of CHOX with an elevated hydrogen concentration differs from the previous testing with another 0.3 wt % Pd/Al_2_O_3_ catalyst (GU-2) which, in contrast, yielded detectable quantities of CHOX for temperatures up to 140 °C at this hydrogen loading.^5^ Two contributory factors for this variance are proposed: (i) WHSV’s varied between the data sets (0.20 h^–1^ here vs 0.46 h^–1^ previously) and (ii) the Pd particle sizes of GU-2 and GU-4 differ (4.0 ± 0.1 vs 3.0 ± 0.3 nm, respectively).

## 4 Discussion

Consideration of catalytic outputs observed for GU-3 (the undiluted 1 wt % Pd parent catalyst) and GU-4 (the 0.3 wt % Pd diluted catalyst) illustrates the comparable chemistry associated with these catalysts; NB conversion (Figure 3), ANL selectivity (Figure 3), and by-product selectivity (Figures 4–6) trends are completely coincident. Exact selectivity values do vary between GU-3 and GU-4 with the latter exhibiting lower by-product selectivity, and associatively, a higher ANL selectivity; however, this observation is to be expected—the lower Pd loading of GU-4 acts to minimize ANL overhydrogenation compared to the higher loading GU-3. Increased ANL selectivity for lower metal loading catalysts has previously been established.^5^ These outcomes indicate that the alumina used to dilute the 1 wt % reference catalyst (GU-3) is not unduly perturbing the observed surface chemistry.

**Scheme 3:**
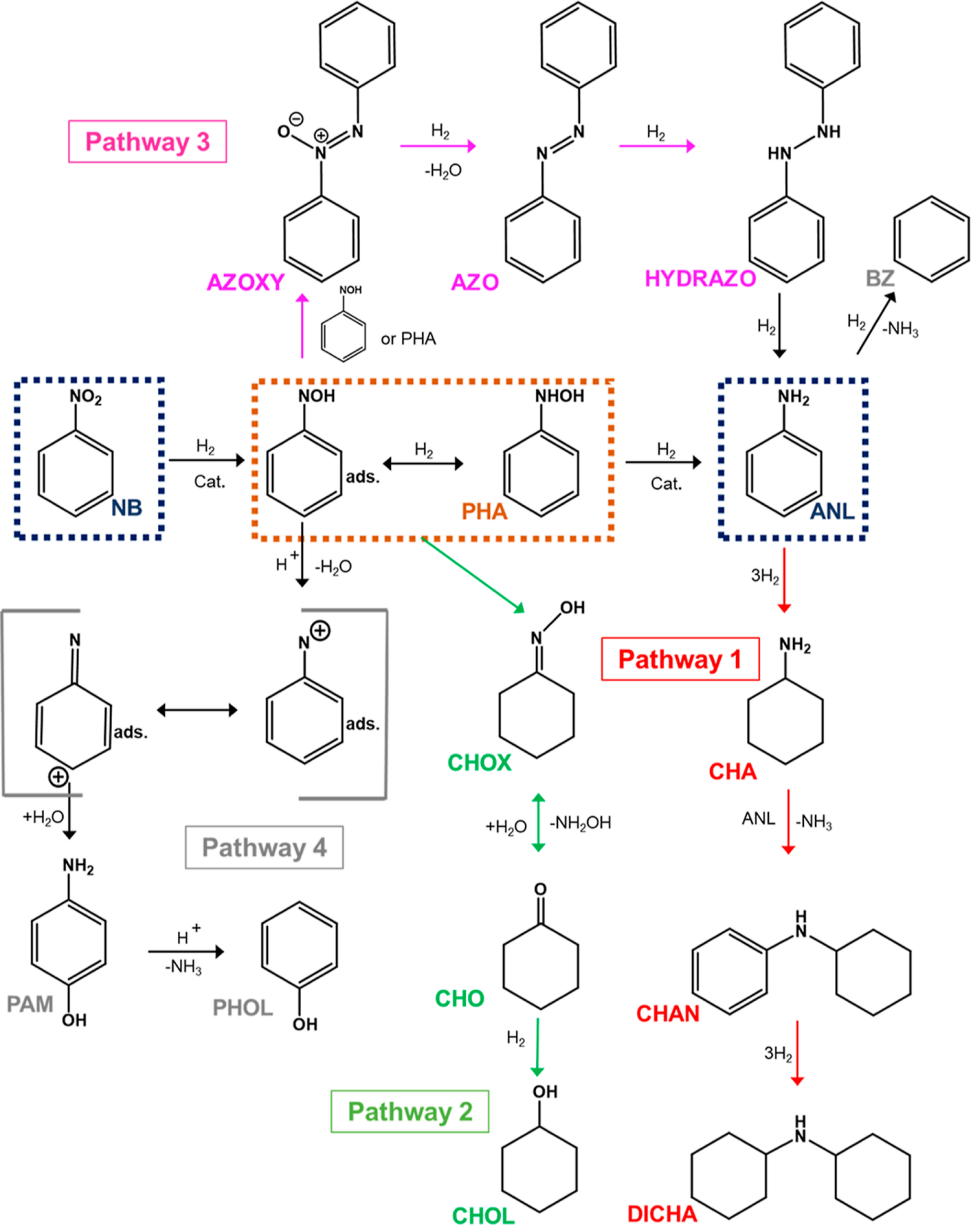
Proposed Mechanism for NB Hydrogenation to ANL via a PHA Intermediate in the Vapor Phase Depicting 4 Pathways to By-product Formation Pathway 1 by-products (red): CHA, CHAN, and DICHA. Pathway 2 by-products (green): CHOX, CHO, and CHOL. Pathway 3 by-products (purple): AZOXY, AZO, and HYDRAZO. Pathway 4 by-products (grey): PAM and PHOL.

Scheme 3 presents a proposed NB hydrogenation scheme depicting the inclusion of CHOX derived from NB intermediates and by-product formation via 4 separate pathways. The principal route is NB hydrogenation to ANL via a series of intermediates. No azo compounds, AZOXY, AZO, or HYDRAZO (Scheme 3, Pathway 3, purple), were detected during the vapor phase testing presented here. However, this route is a well-established mechanism over various catalysts,^7,8,12^ and therefore, it is included within Scheme 3 for completeness. Moreover, it is noted that we have observed AZO formation over Pd/Al_2_O_3_ (GU-2) under specific conditions reported elsewhere.^5^ Another candidate for inclusion in Scheme 3 is ANIL, which is reported in liquid phase studies.^18,37,38^ ANIL formation is proposed to arise via coupling between CHO and ANL,^16,18^ or from a combination of CHA and ANL.^37^ However, as ANIL is not observed in the vapor phase set-up utilized for this investigation, it is excluded from Scheme 3.

As stated in Section 3.2.1, pathway 1 is the by-product pathway arising from the overhydrogenation of ANL that results in CHA, CHAN, and DICHA production.^5^ Literature reports two separate pathways for the production of CHA versus CHAN and DICHA from an amine intermediate derived from ANL: (i) the amine intermediate is hydrogenated to give CHA,^16,18^ and (ii) the amine intermediate combines with ANL to yield a phenylamine intermediate which undergoes subsequent hydrogenation to CHAN and then DICHA.^16^ For Scheme 3, the formation of CHA is simply presented as a ring hydrogenation step, which forms part of a consecutive pathway to CHAN and DICHA (Scheme 3, pathway 1, red), with DICHA as the thermodynamic minimum. The authors recognize the possibility of additional DICHA formation via a condensation reaction between CHA and pathway 2 by-product CHOL; however, as pathway 2 by-products were identified in very low quantities here, this route to DICHA formation is believed to be negligible, if it occurs at all, and, thus, has not been included in Scheme 3.

**Scheme 4:**
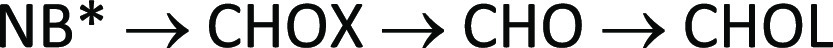
Proposed Pathway for Formation of CHOX, CHO, and CHOL from NB-Derived Intermediates (NB*) (Pathway-2)

Pathway 2 (Scheme 3, green) is proposed to arise from NB-derived intermediates, yielding CHOX, which then undergoes further transformation to CHO and finally is hydrogenated to CHOL (Scheme 4).

Whilst a comprehensive kinetic study of Schemes 2 and 4 is beyond the scope of the present communication, it is acknowledged that precedents exist for CHOX participation in related chemical pathways. Corma et al. established that hydrogenation of NB to CHOX was possible with 97% yield using a one-pot reaction catalyzed by bifunctional Pd/C and Au/C catalysts that included the addition of hydroxylamine hydrochloride.^37^ They proposed that CHOX was derived from the hydrolysis of ANIL to CHO and ANL, with the reaction between CHO and hydroxylamine hydrochloride producing CHOX. CHOX formation from the liquid-phase reaction between CHO and hydroxylamine is reported throughout the literature,^37,39,40^ with hydroxylamine consistently added as a reagent. For this investigation hydroxylamine is absent from the feedstock; thus, this route is not applicable to the work presented here.

Investigations by Egberink and Van Heerden^40^ and Jencks^41^ report the formation of CHOX and H_2_O from the reaction between CHO and hydroxylamine to be a reversible process in a homogeneous system, with the dehydration of CHOX to CHO and hydroxylamine reported to be acid-catalyzed via protonation of CHOX. On this basis, we postulate that CHO is likely derived from CHOX, with subsequent hydrogenation producing CHOL (Scheme 4). The authors would like to note that the hydrolysis of CHOX to CHO would thus introduce hydroxylamine to the reaction mixture; however, derivation of hydroxylamine is only possible post-CHOX formation. As a result, the presence of this hydroxylamine in the reaction mixture cannot be utilized to rationalize initial CHOX formation.

Additionally, during numerous NB hydrogenation reactions, inconsistent observations of phenol (PHOL) were noted. Quartarone et al. investigated the mechanism of liquid phase NB hydrogenation to *para*-aminophenol (PAM) with a range of carbon-supported metal catalysts (Pd/C, Pt/C, and Ru/C) using CH_3_CN–H_2_O–CF_3_COOH as a solvent.^42^ Upon adsorption to the Pt/C catalyst, the resulting Pt-hydroxy amino intermediate yielded a series of surface-adsorbed nitrenium ions which, after the nucleophilic attack of H_2_O, formed PAM.^42^ We suggest that the Pd–hydroxyamino intermediate^5,12^ and water, formed from NB hydrogenation, engage in similar chemistry to produce PAM via the same route. Subsequent decomposition of PAM would yield PHOL and ammonia (Scheme 3, pathway 4 (grey)) and may rationalize the authors’ sporadic observation of PHOL during reaction testing. Quartarone et al. additionally rationalized the formation of PAM in the liquid phase via a Bamberger rearrangement of the intermediate PHA. In our vapor phase studies, PHA^7,8^ may additionally combine with CHO in a similar fashion to that reported for CHO and hydroxylamine,^41,42^ resulting in the formation of CHOX and PHOL.

In summary, Scheme 3 provides a comprehensive description of the principal chemistry associated with NB hydrogenation over low-loading Pd/γ-Al_2_O_3_ catalysts that are capable of operating at elevated temperatures compatible with possible heat recovery scenarios connected with large-scale ANL production. The reactivity of Pd/γ-Al_2_O_3_ catalysts for NB hydrogenation to ANL has previously been evaluated, with a range of by-products reported and temperature identified as a key operational parameter affecting product selectivity.^5,15,16,38^ Here, we report similar observations but with the unique identification of CHOX as a by-product during low metal loading catalysis and a constrained hydrogen supply. Moreover, the approach of undertaking reaction testing procedures over a 0.3 wt % Pd/γ-Al_2_O_3_ catalyst derived from a higher Pd loading reference material has enabled deductions on the Pd crystallite morphology of the lower loading sample, which defines the catalytic platform, to be assessed. This then provides the opportunity to account for the chemistry observed to be inherently linked to a more tightly specified catalyst formulation than was previously the case. Indeed, the concept of site-selective chemistry for NB hydrogenation over low loading Pd/Al_2_O_3_ catalysts constitutes a “work in progress”.

## 5 Conclusions

Two Pd/γ-Al_2_O_3_ catalysts [Pd loadings of 1.0 (GU-3) and 0.3 (GU-4) wt %] have been examined for the vapor phase hydrogenation of NB over the temperature range of 60–200 °C. Most of the reaction testing was undertaken at a H_2_/C_6_H_5_NO_2_ molar flow ratio of 40:1; however, a small number of measurements were undertaken under conditions of a significant hydrogen excess. The 0.3 wt % Pd catalyst is derived from the 1.0 wt % Pd sample. The following conclusions can be drawn.

- CHOX is identified as a by-product that is associated with reagent transformation.
- The morphology of the Pd crystallites of the 0.3 wt % Pd/γ-Al_2_O_3_ catalyst (GU-4) has been inferred with reference to TP-IR and TPD measurements of chemisorbed CO on GU-3.
- The lower Pd loading sample (GU-4) exhibits a higher ANL selectivity by virtue of the minimization of product overhydrogenation.
- Studies that were undertaken employing elevated hydrogen flow rates lead to the proposition of consecutive reagent (Scheme 4)- and product (Scheme 2)-derived by-product formation pathways.
- A global reaction scheme is proposed (Scheme 3) that defines the by-product distribution accessible by the grades of catalyst examined. This information is helpful in defining product purification procedures that would be required in certain heat recovery scenarios connected with large-scale ANL production.

## Supporting Information Available

Glossary of terms, schematic representation of the reaction test apparatus, XRD diffraction patterns for GU-3 and γ-Al_2_O_3_, terminology used to describe the Pd/Al_2_O_3_ catalysts examined in this study and in two preceding publications,^5,21^ characterization of GU-5, selectivity profile for NB hydrogenation over GU-5 as a function of temperature, GC–MS spectrum of the “unknown” by-product, assignment of mass fragments of the GC–MS spectrum of the “unknown” by-product, and NB conversion and ANL selectivity as a function of reaction temperature for GU-3 and GU-4 under conditions of excess hydrogen flow (PDF)

## References

[ref1] Kahl T.; Schröder K. W.; Lawrence F.; Marshall W.; Höke H.; Jäckh R. Aniline. In Ullmann’s Encyclopedia of Industrial Chemistry; Ley C., Elvers B., Eds.; Wiley: Weinheim, 2012; pp 45–478.

[ref2] Brereton G. Polyurethanes. In Ullmann’s Encyclopedia of Industrial Chemistry; Ley C., Elvers B., Eds.; Wiley: Weinheim, 2019; pp 1–76.

[ref3] NIST Chemistry WebBook. NIST Standard Reference Database Number 69. NIST, 2021 (accessed Nov 2, 2021).

[ref4] Randall D.; Lee S. The Polyurethanes Book; John Wiley & Sons: New York, 2002.

[ref5] Morisse C. G. A.; McCullagh A. M.; Campbell J. W.; How C.; MacLaren D. A.; Carr R. H.; Mitchell C. J.; Lennon D. Toward high selectivity aniline synthesis catalysis at elevated temperatures. Ind. Eng. Chem. Res. 2021, 60, 17917–17927. 10.1021/acs.iecr.1c03695. 35115738 PMC8802303

[ref6] Päβler F.; Freund H.-J. Model-based design of energy efficient reactors. Chem. Ing. Tech. 2018, 90, 852–863. 10.1002/cite.201700124.

[ref7] Haber F. Über stufenweise redukton des nitrobenzols mit begrenztem kathodenpotential. Z. Elektrochem. Angew. Phys. Chem. 1898, 4, 506 10.1002/bbpc.18980041705.

[ref8] Gelder E. A.; Jackson S. D.; Lok C. M. The hydrogenation of nitrobenzene to aniline: a new mechanism. Chem. Commun. 2005, 4, 522–524. 10.1039/b411603h. 15654390

[ref9] Relvas J.; Andrade R.; Freire F. G.; Lemos F.; Araújo P.; Pinho M. J.; Nunes C. P.; Ribeiro F. R. Liquid phase hydrogenation of nitrobenzene over an industrial Ni/SiO2 supported catalyst. Catal. Today 2008, 133–135, 828–835. 10.1016/j.cattod.2007.11.050.

[ref10] Zhao F.; Ikushima Y.; Arai M. Hydrogenation of nitrobenzene with supported platinum catalysts in supercritical carbon dioxide: effect of pressure, solvent and metal particle size. J. Catal. 2004, 224, 479–483. 10.1016/j.jcat.2004.01.003.

[ref11] Diao S.; Qian W.; Luo G.; Wei F.; Wang Y. Gaseous catalytic hydrogenation of nitrobenzene to aniline in a two-stage fluidized bed reactor. Appl. Catal., A 2005, 286, 30–35. 10.1016/j.apcata.2005.02.026.

[ref12] Zhang L.; Jiang J.; Shi W.; Xia S.; Ni Z.; Xiao X. Insights into the hydrogenation mechanism of nitrobenzene to aniline on Pd3/Pt(111): a density functional theory study. RSC Adv. 2015, 5, 34319 10.1039/c5ra02389k.

[ref13] Narayanan S.; Pillai Unnikrishnan R. Comparison of hydrogen adsorption and aniline hydrogenation over co-precipitated Co/Al2O3 and Ni/Al2O3 catalysts. J. Chem. Soc., Faraday Trans. 1997, 93, 2009–2013. 10.1039/a608074j.

[ref14] Narayanan S.; Unnikrishnan R.; Vishwanathan V. Nickel-alumina prepared by constant and varying pH method: Evaluation by hydrogen-oxygen chemisorption and aniline hydrogenation. Appl. Catal., A 1995, 129, 9–19. 10.1016/0926-860x(95)00087-9.

[ref15] Couto C. S.; Madeira L. M.; Nunes C. P.; Araújo P. Commercial catalysts screening for nitrobenzene hydrogenation. Appl. Catal., A 2016, 522, 152–164. 10.1016/j.apcata.2016.04.032.

[ref16] Couto C. S.; Madeira L. M.; Nunes C. P.; Araújo P. Hydrogenation of nitrobenzene over a Pd/Al2O3 catalyst - Mechanism and effect of the main operating conditions. Chem. Eng. Technol. 2015, 38, 1625–1636. 10.1002/ceat.201400468.

[ref17] Rubio-Marqués P.; Leyva-Pérez A.; Corma A. A bifunctional palladium/acid solid catalyst performs the direct synthesis of cycloxylanilines and dicyclohexylamines from nitrobenzenes. Chem. Commun. 2013, 49, 8160–1862. 10.1039/c3cc44064h 23925659

[ref18] Nagata T.; Watanabe K.; Kono Y.; Tamaki A.; Kobayahsi T. Process for preparing high-purity aniline. U.S. Patent 5,283,365 A, 1994.

[ref19] Greenfield H. Hydrogenation of aniline to cyclohexylamine with platinum metal catalysts. J. Org. Chem. 1964, 29, 3082–3084. 10.1021/jo01033a512.

[ref20] Chatterjee M.; Sato M.; Kawanami H.; Ishizaka T.; Yokoyama T.; Suzuki T. Hydrogenation of aniline to cyclohexylamine in supercritical carbon dioxide: Significance of phase behaviour. Appl. Catal., A 2011, 396, 186–193. 10.1016/j.apcata.2011.02.016.

[ref21] McCullagh A. M.; Warringham R.; Morisse C. G. A.; Gilpin L. F.; Brennan C.; Mitchell C. J.; Lennon D. A comparison of experimental procedures for the application of infrared spectroscopy to probe the surface morphology of an alumina supported palladium catalyst. Top. Catal. 2021, 64, 1010–1020. 10.1007/s11244-021-01435-y.

[ref22] Chauruka S. R.; Hassanpour A.; Brydson R.; Roberts K. J.; Ghadiri M.; Stitt H. Effect of mill type on the size reduction and phase transformation of gamma alumina. Chem. Eng. Sci. 2015, 134, 774–783. 10.1016/j.ces.2015.06.004.

[ref23] Lundie D. T. Investigation of the Active Sites on Methyl Chloride Synthesis Catalysts. Ph. D. Dissertation, University of Glasgow, U.K., 2003.

[ref24] Lennon D.; Marshall R.; Webb G.; Jackson S. D. The effect of hydrogen concentration on propyne hydrogenation over a carbon supported palladium catalyst studied under continuous flow conditions. Stud. Surf. Sci. Catal. 2000, 130, 245 10.1016/s0167-2991(00)80964-7.

[ref25] Weissermal K.; Arpe H.-J. Industrial Organic Chemistry, 4th ed; Wiley: Heppenheim, 2003; pp 449–450.

[ref26] Iulianelli A.; Longo T.; Basile A. Methanol steam reforming in a dense Pd-Ag membrance reactor: The pressure and WHSV effects on CO-free H2 production. J. Membr. Sci. 2008, 323, 235–240. 10.1016/j.memsci.2008.05.021.

[ref27] Wan Z.; Li G.; Wang C.; Yang H.; Zhang D. Effect of reaction conditions on methanol to gasoline conversion over nanocrystal ZSM-5 zeolite. Catal 2018, 314, 107–113. 10.1016/j.cattod.2018.01.017.

[ref28] Tran Q. K.; Han S.; Ly H. V.; Kim S.-S.; Kim J. Hydrodeoxygenation of a bio-oil model compound derived from woody biomass using spray-pyrolysis-derived spherical γ-Al2O3-SiO2 catalysts. J. Ind. Eng. Chem. 2020, 92, 243–251. 10.1016/j.jiec.2020.09.012.

[ref29] Goldsmith D.; Becher D.; Sample S.; Djerassi C. Mass spectrometry in structural and stereochemical problems-XCVII: A study of the fragmentation processes of oximes. Tetrahedron 1966, 22, 145–173. 10.1016/S0040-4020(01)99103-3.

[ref30] Lear T.; Marshall R.; Antonio Lopez-Sanchez J.; Jackson S. D.; Klapötke T. M.; Bäumer M.; Rupprechter G.; Freund H.-J.; Lennon D. The application of infrared spectroscopy to probe the surface morphology of alumina-supported palladium catalysts. J. Chem. Phys. 2005, 123, 174706 10.1063/1.2101487. 16375556

[ref31] Hollins P. Effects of dipolar coupling on the intensity of infrared absorption bands from supported metal catalysts. Spectrochim. Acta, Part A 1987, 43, 1539–1542. 10.1016/s0584-8539(87)80044-2.

[ref32] Dai C.; Li Y.; Ning C.; Zhang W.; Wang X. The influence of alumina phases on the performance of Pd/Al2O3 catalyst in selective hydrogenation of benzonitrile to benzylamine. Appl. Catal., A 2017, 545, 97–103. 10.1016/j.apcata.2017.07.032.

[ref33] Sandoval V. H.; Gigola C. E. Characterisation of Pd and Pd-Pb/α-Al2O3 catalysts. A TPR—TPD study. Appl. Catal., A 1996, 148, 81–96. 10.1016/s0926-860x(96)00224-4.

[ref34] Dularent O.; Chandes K.; Bouly C.; Bianchi D. Heat of adsorption of carbon monoxide on a Pd/Al2O3 solid using in situ infrared spectroscopy at high temperatures. J. Catal. 1999, 188, 237–251. 10.1006/jcat.1999.2661.

[ref35] Monteiro R. S.; Dieguez L. C.; Schmal M. The role of Pd precursors in the oxidation of carbon monoxide over Pd/Al2O3 and Pd/CeO2/Al2O3 catalysts. Catal 2001, 65, 77–89. 10.1016/s0920-5861(00)00547-2.

[ref36] Lear T.; Hamilton N. G.; Lennon D. The application of temperature-programmed desorption, adsorption isotherms and temperature-programmed oxidation to investigate the interaction of CO with alumina-supported palladium catalysts. Catal. Today 2005, 126, 219–227. 10.1016/j.cattod.2007.03.014.

[ref37] Rubio-Marqués P.; Hernández-Garrido J. C.; Leyva-Pérez A.; Corma A. One pot synthesis of cyclohexanone oxime from nitrobenzene using a bifunctional catalyst. Chem. Commun. 2014, 60, 1645–1647. 10.1039/C3CC47693F. 24270690

[ref38] Couto C. S.; Madeira L. M.; Nunes C. P.; Araújo P. Liquid-phase hydrogenation of nitrobenzene in a tubular reactor: parametric study of operation conditions influence. Ind. Eng. Chem. Res. 2017, 56, 3231–3242. 10.1021/acs.iecr.7b00403.

[ref39] Pietrobon L.; Ronchin L.; Sadraoui C.; Pontello R.; Tosetto C.; Vavasori A. Pd/C catalysed selective hydrogenation of nitrobenene to cyclohexanone oxime in the presence of NH2OH.HCl: Influence of the operative variables and insights on the reaction mechanism. Appl. Catal., A 2020, 598, 117570 10.1016/j.apcata.2020.117570.

[ref40] Egberink H.; Van Heerden C. The mechanism of the formation and hydrolysis of cyclohexanone oxime in aqueous solutions. Anal. Chim. Acta 1980, 118, 359–368. 10.1016/s0003-2670(01)93612-8.

[ref41] Jencks W. P. Studies on the mechanism of oxime and semicarbazone formation. J. Am. Chem. Soc. 1959, 81, 475–481. 10.1021/ja01511a053.

[ref42] Quartarone G.; Ronchin L.; Tosetto A.; Vavasori A. New insight on the mechanism of the catalytic hydrogenation of nitrobenzene to 4-aminophenol in CH3CN-H2O-CF3COOH as a resuable solvent system. Hydrogenation of nitrobenzne catalysed by precious metals supported on carbon. Appl. Catal., A 2014, 475, 169–178. 10.1016/j.apcata.2014.01.033.

