# Supplementary material for: Mechanistic Insight Into the Application of Alumina-Supported Pd Catalysts for the Hydrogenation of Nitrobenzene to Aniline
Source: Ind Eng Chem Res. 2022 Jul 14;61(30):10712–22. doi: 10.1021/acs.iecr.2c01134 (PMC9354085; doi:10.1021/acs.iecr.2c01134)
Supplement: Supplementary file 1 — ie2c01134_si_001.pdf [file ie2c01134_si_001.pdf]

Supporting Information

Mechanistic insight into the application of alumina-supported Pd catalysts for the hydrogenation of nitrobenzene to aniline

C.G.A. Morisse[1], A.M. McCullagh[1], J.W. Campbell[1], C.J. Mitchell[2], R. Carr[3] and D. Lennon*[1]

1. School of Chemistry, Joseph Black Building, University of Glasgow, Glasgow, G12 8QQ, UK.
2. SABIC UK Petrochemicals Ltd., The Wilton Centre, Redcar, TS10 4RF, UK.
3. Huntsman Polyurethanes, Everslaan 45, 3078 Everberg, Belgium.

Glossary of Terms. Lists abbreviations and full nomenclature for discussed chemical compounds.

Figure S1. Schematic representation of the reaction test apparatus.

Figure S2. XRD diffraction patterns for (a) GU-3 and (b) the γ-alumina utilised for the preparation of GU-4 via dilution. The red lines indicate 2θ values for γ-alumina.

Table S1. Terminology used to describe (i) the two catalysts explicitly examined in this study (GU-3 and GU-4), including (ii) the catalyst used to produce quantifiable quantities of cyclohexanone oxime (GU-5), and (iii) the two catalysts examined in two preceding publications (GU-1 and GU-2)

Table S2. Characterisation of GU-5 (0.3 wt % Pd/Al_2O_3 technical grade egg-shell catalyst supplied by Huntsman Polyurethanes [ASC-2]): Metal loading, surface area, uptake of CO, metal dispersion, particle size and concentration of surface Pd atoms.

Figure S3. Selectivity profile for by-product formation for nitrobenzene hydrogenation over GU-5 as a function of temperature. Prior to commencing the temperature ramp, the catalyst had experienced a 16 h conditioning period at 60 °C.

Figure S4. GC-MS spectrum of 'unknown' by-product obtained via isolation of product stream from nitrobenzene hydrogenation over GU-5 at 60 °C.

Table S3. Assignment of mass fragments observed in the GC-MS spectrum of the 'unknown' compound obtained from the reaction mixture of nitrobenzene hydrogenation over GU-5 at 60 °C (Figure S4).

Figure S5. Nitrobenzene conversion (grey) and aniline selectivity (red) as a function of reaction temperature in the presence of an enhanced flow of dihydrogen (H_2:$C_6H_5NO_2$ molar flow ratio = 600:1, WHSV = 0.20 h^{-1}): (a) GU-3 and (b) GU-4.

Glossary of Terms

NB:	nitrobenzene
NSOB:	nitrosobenzene
PHA:	phenylhydroxylamine
ANL:	aniline
AZOXY:	azoxybenzene
AZO:	azobenzene
HYDRAZO:	hydrazobenzene
CHA:	cyclohexylamine
CHO:	cyclohexanone
NPHA:	N-[1-(amino)cyclohexyl]-N-phenylamine
CHAN:	N-cyclohexylaniline
ANIL:	N-cyclohexylidenaniline
BZ:	benzene
CHOL:	cyclohexanol
DICHA:	dicyclohexylamine
CHOX:	cyclohexanone oxime
PAM:	para-aminophenol
PHOL:	phenol

Experimental Set-Up

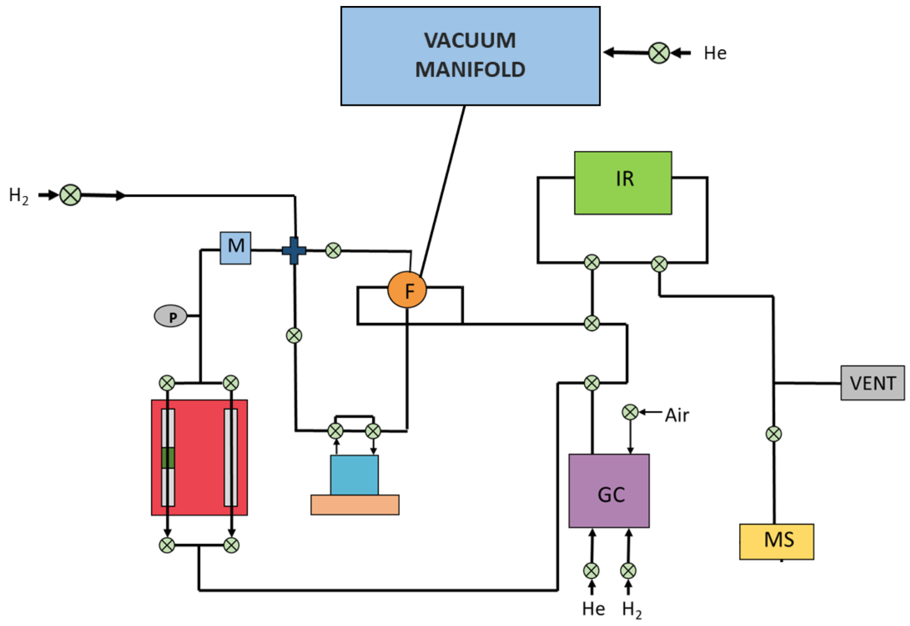

Figure S1. Schematic representation of the reaction test apparatus.

The apparatus permits both catalyst characterisation investigations (CO TP-IR, TPD) and collection of reaction testing data. Figure S1 presents a schematic representation of this set-up with black lines depicting connections made with 1/8" stainless-steel Swagelok tubing. The experimental set-up consists of a custom-built glass-line utilised as a vacuum manifold, a 1/4-inch stainless-steel continuous plug flow reactor (I.D.: 0.18"), a PID temperature control system, mass flow-controlled gas supply arrangements, a nitrobenzene delivery system and 3 on-line analysis/sampling systems.

The diffusion pumped glass-line permits controlled pulses of CO to be introduced to the apparatus. Pressure monitoring (capacitance manometer) permits quantification of pulsed CO doses to the catalyst. Vapour phase nitrobenzene is introduced to the apparatus via the controlled heating of a temperature-controlled glass bubbler. He is utilised as a carrier gas. All Swagelok tubing utilised for the transport of nitrobenzene throughout the system is maintained at 60 °C with heating tape (Electrothermal) to retain the reagent in the vapour phase; this is not depicted schematically in Figure S1. An in-line gas purification system (Agilent) is integrated into the He lines prior to the mass flow controller to remove any trace levels of moisture and oxygen present before the diluent flow is introduced to the reaction system.

A series of Swagelok ball-valves and Young's taps permit interchangeable flow path-ways through the apparatus, permitting access to the three on-line methods of analysis for catalyst investigations: infrared spectroscopy, mass spectrometry and gas-liquid chromatography. The accessibility of clean pulses of CO to the set-up and the Nicolet Nexus infrared spectrometer fitted with a SpectraTech smart diffuse reflectance cell and environmental chamber permit in situ catalyst morphology investigations utilising CO as a probe molecule over supported metal catalysts. One should note that the above set-up is utilised in an in situ capacity for the present investigation, however with the accessibility of the mass spectrometer the line does provide *operando* capability. Owed to the engineering of the discussed experimental set-up, it is possible to direct CO pulses to (i) the infrared cell or (ii) the reactor, permitting CO TPD measurements via utilisation of the mass spectrometer (MKS Microvision Plus). Comparison of data derived via CO TP-IR and CO TPD measurements permits a correlation between CO desorption trends observed in the IR cell with those observed in the same reactor utilised for reaction testing data, and thereby allows for interrelationships between reaction testing data and IR inferred catalyst morphology to be investigated.

For reaction testing nitrobenzene and hydrogen are mixed in a gas mixing vessel (Figure S1, M) prior to introduction to the reactor; the eluting gases are analysed via GLC (Agilent 6850 series II) fitted with an FID detector and utilising an automated gas sampling valve (250 μL).

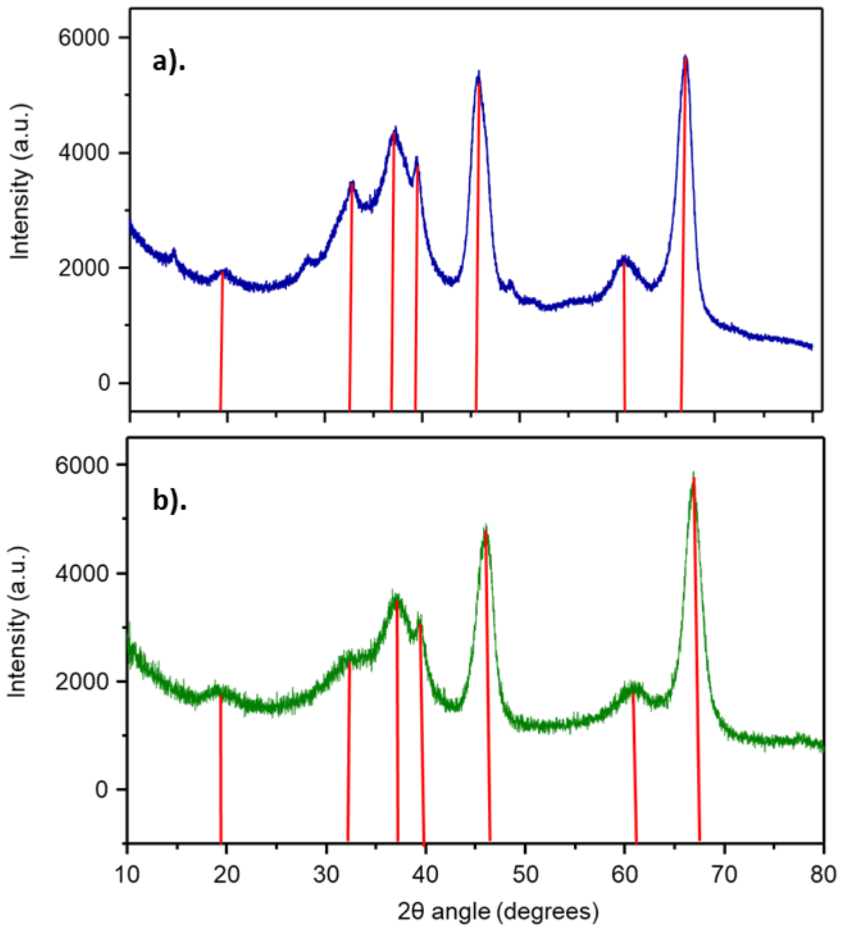

Figure S2. XRD diffraction patterns for (a) GU-3 and (b) the γ-alumina utilised for the preparation of GU-4 via dilution. The red lines indicate 2θ values for γ-alumina.

Table S1. Terminology used to describe (i) the two catalysts explicitly examined in this study (GU-3 and GU-4), including (ii) the catalyst used to produce quantifiable quantities of cyclohexanone oxime (GU-5), and (iii) the two catalysts examined in two preceding publications (GU-1 and GU-2).

Catalyst descriptor	Supplier	Code adopted within articles	Coding for technical grade catalysts	Reference
**5 wt % Pd/γ-Al_2O_3**	Alfa Aesar	GU-1		[5], [20]
**0.3 wt % Pd/Al_2O_3**	Huntsman Polyurethanes	GU-2	ASC-1	[5]
**1 wt % Pd/γ-Al_2O_3**	Alfa Aesar	GU-3		a
**1 wt % Pd/γ-Al_2O_3 diluted to a loading of 0.3 wt % Pd/γ-Al_2O_3**	-	GU-4		a
**0.3 wt % Pd/Al_2O_3**	Huntsman Polyurethanes	GU-5	ASC-2	a

[a] This study.

Table S2. Characterisation of GU-5 (0.3 wt % Pd/Al_2O_3 technical grade egg-shell catalyst supplied by Huntsman Polyurethanes [ASC-2]): Metal loading, surface area, uptake of CO, metal dispersion, particle size and concentration of surface Pd atoms.

Nominal Loading (wt %)	Actual Pd Loading (AAS) (wt %)	BET surface area ($m^2\ g^{-1}$)	Saturation coverage of CO (µmol CO $g^{-1}_{(cat)}$)	Surface Pd atoms (µmol $g_{(cat)}^{-1}$)	Catalyst Dispersion (%)	Calc. Mean Pd Particle size (nm)[1]	Observed Mean Pd Particle Size (TEM) (nm)
**0.3**	0.25	30.28	6.76	13.5	47.9	2.3	~3

[1] Mean particle size calculated from CO adsorption isotherm.

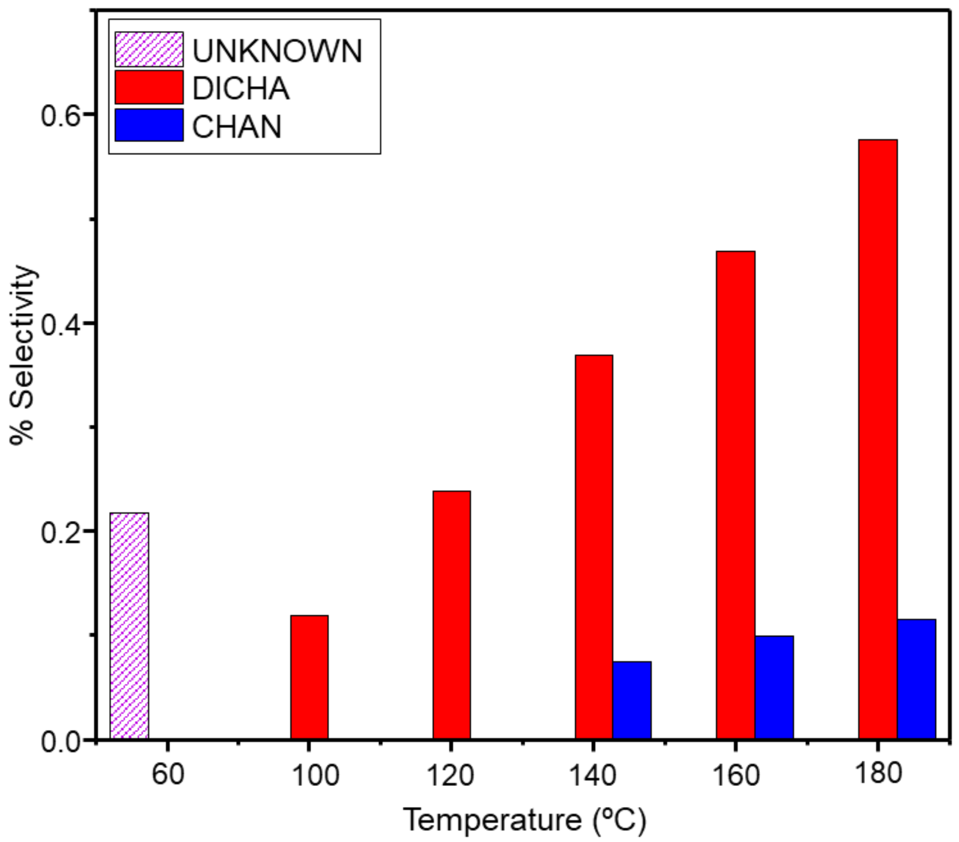

Figure S3. Selectivity profile for by-product formation for nitrobenzene hydrogenation over GU-5 as a function of temperature. Prior to commencing the temperature ramp, the catalyst had experienced a 16 h conditioning period at 60 °C.

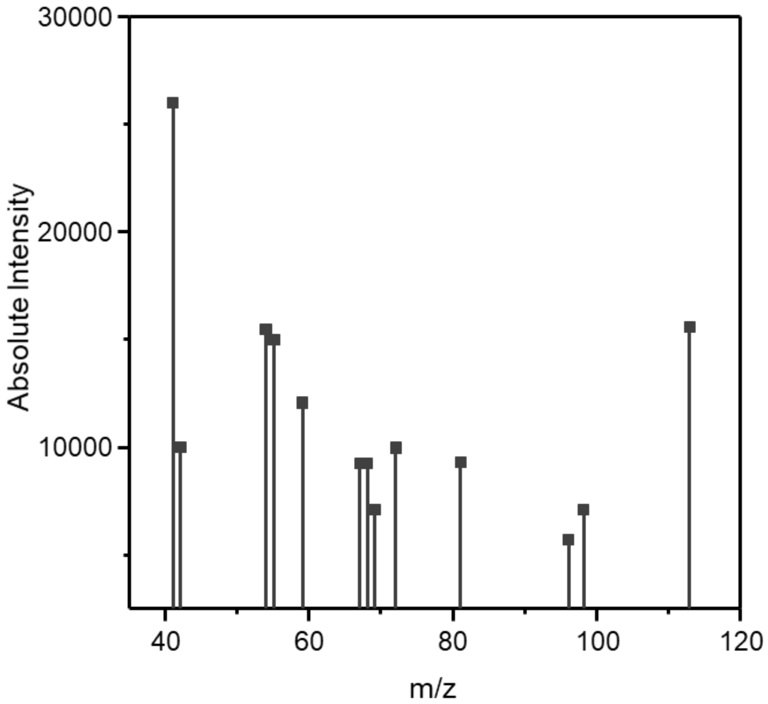

Figure S4. GC-MS spectrum of 'unknown' by-product obtained via isolation of product stream from nitrobenzene hydrogenation over GU-5 at 60 °C.

M/Z	Composition	%	M/Z	Composition	%
**41.1**	C_2H_3N	18	**69.1**	C_4H_7N	4.5
**42.1**	C_2H_4N	6.8	**72.1**	C_4H_8O	6.6
**54.0**	C_3H_4N	11	**81.1**	C_6H_9	4.5
**55.1**	C_4H_7	10	**96.1**	$C_6H_{10}N$	3.5
**59.1**	C_2H_5NO	7.9	**98.2**	$C_6H_{10}O$	4.5
**67.1**	C_4H_5N	6.1	**113.0**	$C_6H_{10}NOH$	11
**68.1**	C_4H_6N	6.1			

Table S3. Assignment of mass fragments observed in the GC-MS spectrum of the 'unknown' compound obtained from the reaction mixture of nitrobenzene hydrogenation over GU-5 at 60 °C (Figure S4).

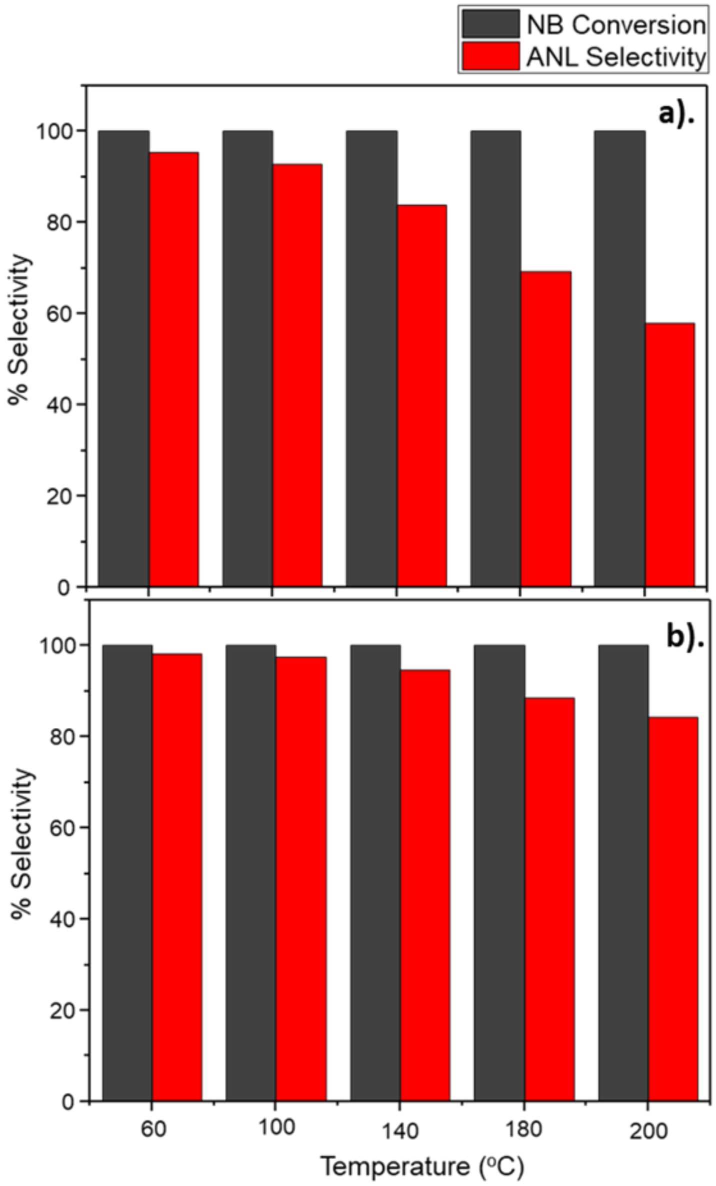

Figure S5. Nitrobenzene conversion (grey) and aniline selectivity (red) as a function of reaction temperature in the presence of an enhanced flow of dihydrogen (H_2:$C_6H_5NO_2$ molar flow ratio = 600:1, WHSV = 0.20 h^{-1}): (a) GU-3 and (b) GU-4.
